# A reconstruction problem for a class of phylogenetic networks with lateral gene transfers

**DOI:** 10.1186/s13015-015-0059-z

**Published:** 2015-12-02

**Authors:** Gabriel Cardona, Joan Carles Pons, Francesc Rosselló

**Affiliations:** Department of Mathematics and Computer Science, University of the Balearic Islands, 07122 Palma de Mallorca, Spain

**Keywords:** Phylogenetic network, Lateral gene transfer, Horizontal gene transfer, Phylogenetic tree

## Abstract

**Background:**

Lateral, or Horizontal, Gene Transfers are a type of asymmetric evolutionary events where genetic material is transferred from one species to another. In this paper we consider *LGT networks*, a general model of phylogenetic networks with lateral gene transfers which consist, roughly, of a *principal* rooted tree with its leaves labelled on a set of taxa, and a set of extra *secondary* arcs between nodes in this tree representing lateral gene transfers. An LGT network gives rise in a natural way to a *principal phylogenetic subtree* and a set of *secondary phylogenetic subtrees*, which, roughly, represent, respectively, the main line of evolution of most genes and the secondary lines of evolution through lateral gene transfers.

**Results:**

We introduce a set of simple conditions on an LGT network that guarantee that its principal and secondary phylogenetic subtrees are pairwise different and that these subtrees determine, up to isomorphism, the LGT network. We then give an algorithm that, given a set of pairwise different phylogenetic trees $T_0,T_1,\ldots ,T_k$ on the same set of taxa, outputs, when it exists, the LGT network that satisfies these conditions and such that its principal phylogenetic tree is $T_0$ and its secondary phylogenetic trees are $T_1,\ldots ,T_k$.

**Electronic supplementary material:**

The online version of this article (doi:10.1186/s13015-015-0059-z) contains supplementary material, which is available to authorized users.

## Background

In the traditional view of evolution, species evolve in a pattern ideally represented by a series of bifurcations in a tree. However, it is well known that many relevant evolutionary processes cannot be properly represented in a tree [1, 2]. This has motivated the adoption, since as early as the second half of the XVIIIth century, of more general models to represent phylogenies [3]. One specific type of non tree-like events are the *Lateral*, or *Horizontal*, *Gene Transfers*: transfers of genetic material from one species to a different and, usually, taxonomically distant one [4]. Although these kinds of phenomena are known since the 1950s [5, 6], the current explosion of genomic and metagenomic data has revealed that they are much more frequent and important than previously thought, not only among unicellular species [7] but also, for instance, among plants [8] or from parasites to hosts [9].

Evolutionary histories including non-tree like events are usually modelled by means of (*evolutionary*) *phylogenetic networks* [10, 11]: rooted directed acyclic graphs with leaves bijectively labelled by a set of taxa. The study of phylogenetic networks has been an active field of research during recent years, as witnessed in [12], and many papers on the computational inference of phylogenetic networks with lateral gene transfer events from incongruent gene trees have been published: see, for instance [13–17].

Although lateral gene transfers are modeled in these papers as arcs added to a tree, and hence the resulting phylogenetic networks are *tree-based* in the sense of [18], in most cases the mathematical model under consideration makes no reference to the base tree and all parents of a node are treated symmetrically. This is not accurate, because in lateral gene transfers, the resulting species acquires its DNA mostly from one, and only one, of its parents, which should be understood as its “principal” parent, in contrast to the other parents which contribute in a much lesser way and should be considered as “secondary” parents. This asymmetry is usually emphasized in graphical representations of phylogenetic networks with lateral gene transfers, like for instance those depicted in [19, Fig. 3] (which, according to Morrison [20], are the first published in the literature), but again seldom in the mathematical model. Actually, and up to our knowledge, the only types of phylogenetic networks that explicitly distinguish between the primary, tree-like, line of evolution and the secondary lateral gene transfers that have been studied in the literature are those in [18] and those in [21, 22]. In [18] the primary line of evolution is given by choosing a *base tree*, but they are not interested in a reconstruction problem from a set of trees but in deciding whether this base tree exists or not for a given phylogenetic network. Also, Górecki’s introduces *species graphs* in [21, 22], although this author was not interested in the reconstruction of phylogenies but in modelling the evolution of genes in the context of the evolution of species.

**Fig. 1:**
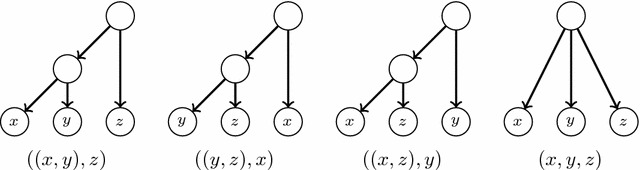
Triples on *x*, *y*, *z*

**Fig. 2:**
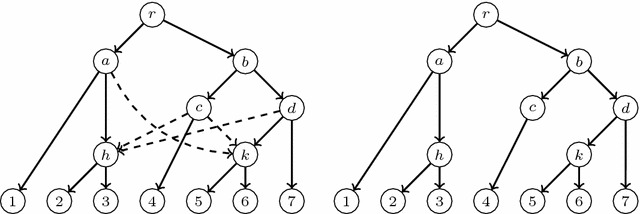
An LGT network (*left*) and its principal subtree (*right*)

**Fig. 3:**
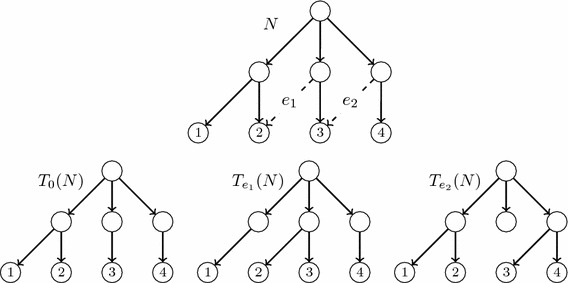
An LGT network, its principal subtree and its secondary subtrees

**Fig. 4:**
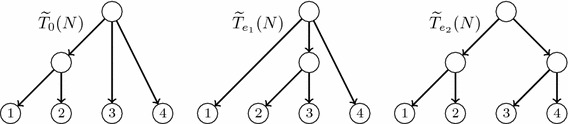
The reduced principal subtree and the reduced secondary subtrees of the LGT network *N* depicted in Fig. 3

**Fig. 5:**
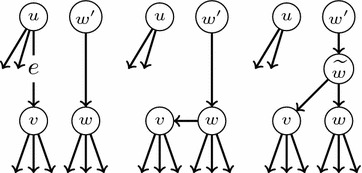
The original tree (*left*), the tree obtained by means of the node rSPR operation $v \mathop {\longleftarrow }\limits ^{{node}}w$ (*middle*), and the tree obtained by means of the arc rSPR operation $v \mathop {\longleftarrow }\limits ^{{arc}}w$ (*right*)

In this paper we consider a general model of phylogenetic network with lateral gene transfers similar to the species graphs’ approach: *LGT networks*, which consist roughly of a *principal* rooted tree with its leaves labelled on a set of taxa (and possibly with *elementary*, that is, out-degree 1, nodes) and a set of *secondary* arcs between nodes in this tree, representing lateral gene transfers, such that the resulting directed graph turns out to be rooted, acyclic, with its leaves labelled and its internal nodes unlabelled. Any such LGT network gives rise to a *principal phylogenetic subtree* (by suppressing out-degree 1 nodes in the principal subtree) and a set of *secondary phylogenetic subtrees*, each one of them obtained by replacing one arc in the principal subtree by one secondary arc with the same target node (and then recursively removing non-labelled leaves and out-degree 1 nodes). These phylogenetic subtrees can be understood, respectively, as representing the primary line of evolution and the secondary histories, involving one lateral gene transfer event.

We then introduce the subclass of *restricted* LGT networks, which are characterized by a set of conditions that guarantee that its principal and secondary phylogenetic subtrees are pairwise different and that these trees determine, up to isomorphism, the LGT network. We also give an algorithm that solves the corresponding reconstruction problem from incongruent trees: given a set of pairwise different phylogenetic trees $T_0,T_1,\ldots ,T_k$ on the same set of taxa, to find, when it exists, the unique restricted LGT network such that its principal phylogenetic tree is $T_0$ and its secondary phylogenetic trees are $T_1,\ldots ,T_k$. In order to test the models and algorithms introduced in this paper, we include a computational experiment on the database of phylogenetic trees given in [23].

## Preliminaries

Let $N=(V,E)$ be a directed acyclic graph. A node $u\in V$ is a *tree node* if ${{\mathrm{indeg}}}(u)\le 1$, and it is a *reticulation* otherwise. A node *u* is a *root* if ${{\mathrm{indeg}}}(u)=0$, and *N* is *rooted* (it is an *rDAG*, for short) if it has a single root. A node *u* is a *leaf* if ${{\mathrm{outdeg}}}(u)=0$, *internal* if it is not a leaf, and *elementary* if ${{\mathrm{outdeg}}}(u)=1$.

For every $u,v\in V$, if $(u,v)\in E$, we say that *u* is a *parent* of *v* and that *v* is a *child* of *u*. Whenever there exists a (directed) path from *u* to *v*, in symbols $u\!\rightsquigarrow \!{}v$, we say that *u* is an *ancestor* of *v* and that *v* is a *descendant* of *u*: notice in particular that every node is both an ancestor and a descendant of itself. A path $u\!\rightsquigarrow \!{}v$ is *proper* when $u\ne v$ (and then *u* is a *proper ancestor* of *v* and *v* is a *proper descendant* of *u*). A path $u\!\rightsquigarrow \!{}v$ is *elementary* when all its nodes, except at most *v* (but including its origin *u*), are elementary.

A *tree* is an rDAG without reticulations. In particular, trees may contain elementary nodes. Given an elementary node *u* in a tree *T*, in order to *suppress* it we perform the following operation: if *u* is the root, we remove it together with its incident arc; if, otherwise, *u* has parent *w* and child *v*, we remove *u* together with the arcs (*w*, *u*) and (*u*, *v*), and we replace them by an arc (*w*, *v*).

Two paths $u\!\rightsquigarrow \!{}v_1$ and $u\!\rightsquigarrow \!{}v_2$ in a tree *T* are *bifurcating* when they have the same origin and it is their only node in common. Given two nodes *u*, *v* in a tree *T*, their *lowest common ancestor*$LCA_T(u,v)$ is their common ancestor that is a descendant of every other common ancestor of them. If *u*, *v* are not connected by a directed path, then $LCA_T(u,v)$ is characterized by the fact that there exist bifurcating paths $LCA_T(u,v)\!\rightsquigarrow \!{}u$ and $LCA_T(u,v)\!\rightsquigarrow \!{}v$.

Let *S* be henceforth a finite, non-empty set of *labels*; in order to avoid unnecessary discussions of trivial cases, we shall always assume that *S* has more than one element. An *S-rDAG* is an rDAG endowed with a bijection between its set of leaves and *S*. We shall always identify, usually without further notice, each leaf in an *S*-rDAG with its label.

In this paper, by a *phylogenetic network* on *S* we mean an *S*-rDAG without elementary nodes. Notice, in particular, that we forbid in our phylogenetic networks the existence of reticulations with out-degree 1. The reason is that, unlike other interpretations [10, 24, 25, 26], we understand that all nodes in a phylogenetic network represent species: each tree node represents a species produced by mutations from its immediate ancestor, while reticulations represent species that have appeared through “reticulate” events involving the interaction of more than one species. Therefore, an elementary node would represent a species that has only one descendant, and it is impossible to distinguish this ancestor species from its unique descendant through evolutive information only.

An *S-tree* is an *S*-rDAG without reticulations, that is, a tree endowed with a bijection between its set of leaves and *S*. A *phylogenetic tree* on *S* is a phylogenetic network on *S* without reticulations, or, equivalently, an *S*-tree without elementary nodes. Every *S*-tree gives rise to a phylogenetic tree on *S* by suppressing all its elementary nodes.

Given a phylogenetic tree *T* on *S* and a subset $S_0\subseteq S$, the *restriction* of *T* to $S_0$ is the phylogenetic tree $T|_{S_0}$ on $S_0$ obtained by first taking the subtree of *T* supported on all ancestors of the leafs in $S_0$ and then suppressing elementary nodes.

Given an *S*-tree $T=(V,E)$, the *cluster* of a node $u \in V$ is the set $C_T(u)\subseteq S$ of labels of leaves that are descendants of *u*. Let $C(T)=\{C_T(u)\mid u\in V\}$.

A *triple* on three different labels $x,y,z\in S$ is a phylogenetic tree on $\{x,y,z\}$. Figure 1 depicts the only four possible triples on *x*, *y*, *z*, together with their Newick notation.^1^ The triple *defined* by a phylogenetic tree *T* on $x,y,z\in S$ is the restriction of *T* to $\{x,y,z\}$; we shall denote it by $T_{x,y,z}$, and the set of all triples defined by *T* by $\Gamma (T)$.

Two *S*-rDAG on the same set *S* are isomorphic if there exists an isomorphism of directed graphs between them that preserves the leaves’ labels. Recall that two phylogenetic trees on *S* are isomorphic if, and only if, they have the same set of clusters, and also if, and only if, they define the same set of triples [27, Theorems 3.5.2 and 6.4.1]. Actually, the descriptions of a phylogenetic tree *T* on *S* by means of *C*(*T*) and $\Gamma (T)$ are equivalent, through the following result (see, for instance, [28, Lemma 9.1]):

### **Lemma 1**

Let *T* be a phylogenetic tree on *S*. For every $\emptyset \ne C\subseteq S$, $C\in C(T)$ if, and only if, $((c,c{^{\prime }}),x)\in \Gamma (T)$, for every $c,c{^{\prime }}\in C$ and $x\in SP{\setminus }C$.

We shall often make the abuse of language of saying that two *S*-rDAG are *equal* to mean that they are actually isomorphic.

## LGT networks

In [21, 22], Górecki defined a species graph on a set of labels *S* as an *S*-tree endowed with a set of extra arcs, representing lateral gene transfers, that satisfies a set of restrictions motivated by their use in the representation of common evolutionary histories of species and genes. In this section we consider phylogenetic networks with lateral gene transfers more general than species graphs, by imposing only that the graph obtained by adding arcs to the tree is a phylogenetic network. In the next section we shall impose a new set of restrictions that will ensure the uniqueness of the solution of the reconstruction problem considered therein.

### **Definition 1**

An *LGT network* on a set *S* is a phylogenetic network $N=(V,E)$ on *S* together with a partition $E=E_p\sqcup E_s$ of its set of arcs such that $T_0(N)=(V,E_p)$ is an *S*-tree. The arcs in $E_p$ are called *principal*, and those in $E_s$, *secondary*. We shall call $T_0(N)$ the *principal subtree* of *N*.

Figure 2 depicts an LGT network and its principal subtree $T_0(N)$.^2^ It is easy to check that any species graph defines an LGT network. Using some other notations that appear in the literature, we also have that $T_0(N)$ is a switching of *N* [29] (or $T_0(N)$ is displayed by *N* [10]); also, *N* is tree-based and $T_0(N)$ is a distinguished base tree [18].

Let *N* be an LGT network. Since $T_0(N)=(V,E_p)$ is an *S*-tree, every arc in *N* ending in a tree node is principal and the set of arcs ending in each reticulation *h* contains exactly one principal arc: we call its origin the *principal parent* of *h*, and its other parents, *secondary parents*. To ease the notations, we shall also say that the single parent of a tree node is its *principal parent*. We also split the children of every node *v* into *principal* and *secondary*, depending on the type of the arcs going from *v* to them. These definitions can be illustrated in Fig. 2; for instance, the node *a* is the principal parent of *h*, and the nodes *c* and *d* are its secondary parents; also, the leaf 4 is the principal child of *c* and the nodes *h* and *k* are its secondary children.

The rationale behind these definitions is as follows. In an LGT network, nodes represent species. The principal subtree represents the main line of evolution of these species; that is, the genetic material of a species comes mainly from its principal parent, possibly including mutations, while its secondary parents have introduced some genes in the species through lateral gene transfers. In this way, a secondary arc models a lateral gene transfer from its source to the principal parent of its target.

The fact that $T_0(N)$ is an *S*-tree also implies that every internal node of *N* has some principal child. A node *v* is *principally elementary* when it has exactly one principal child, i.e., when it is elementary in $T_0(N)$. Since *N* cannot contain elementary nodes, this implies that every principally elementary node is the source of some secondary arc. A *principally elementary path* in *N* is an elementary path in $T_0(N)$.

A path in an LGT network *N* is *principal* when it consists only of principal arcs. The *principal cluster* of a node *u* is the set $C_{T_0(N)}(u)$ of leaves that are *principal descendants* of *u*; that is, that can be reached from *u* through principal paths.

For each secondary arc $e=(u,h)$ in *N*, the *secondary subtree*$T_e(N)$ of *N* associated to *e* is the tree obtained from $T_0(N)$ by removing the principal arc ending in *h* and replacing it by *e*; cf. Fig. 3. Notice that the tree $T_e(N)$ is also a switching of *N*, and this switching can be obtained from the one associated to $T_0(N)$ by *switching-off* the principal arc ending in *h* and *switching-on* the arc *e*.

Although $T_0(N)$ is always an *S*-tree, a secondary subtree of *N* may have non-labelled leaves: we shall say that it is *partially leaf-labelled in S*. To obtain phylogenetic trees on *S* from the principal and secondary subtrees of *N*, we *reduce* them: we recursively remove (in secondary subtrees) all their non labelled leaves together with the arcs ending in them, and then we recursively suppress all their elementary nodes. We shall generically denote by $\widetilde{T}$ the *reduced phylogenetic tree* on *S* obtained by reducing a partially leaf-labelled tree *T* on *S*. Notice that $\widetilde{T}$ is an *homeomorphic subtree* of *T*, in the sense that they have the same set of labels, the set of nodes of $\widetilde{T}$ is contained in the set of nodes of *T*, this inclusion preserves the leaves’ labelling, and every arc in $\widetilde{T}$ corresponds to a path in *T*. In particular, for every node *v* in $\widetilde{T}$, $C_T(v)=C_{\widetilde{T}}(v)$; we shall often use this equality without any further mention. The construction of the reduced principal and secondary subtrees of an LGT network is illustrated by Figs. 3 and 4.

The following result is a direct consequence of the fact that the set of triples defined by a phylogenetic tree characterizes it, and that the triple defined on a set of three labels by a partially leaf-labelled tree with, possibly, elementary nodes, is the same as the triple defined by its reduction.

### **Proposition 1**

*Let *$T_1,T_2$* be two partially leaf-labelled trees on a setS*. Then, $\widetilde{T}_1= \widetilde{T}_2$* if, and only if,*$T_1$* and *$T_2$* define the same triple on each set of three different labels ofS*.

Intuitively, the difference between the reduced principal subtree $\widetilde{T}_0(N)$ and any reduced secondary subtree $\widetilde{T}_e(N)$ is that some rooted subtree of the former is pruned (by removing the principal arc ending in the end of *e*) and regrafted (through the secondary arc *e*) in the latter. This fact motivates to consider *rooted subtree prune and regraft* (*rSPR*, for short) operations [30] to analyze the differences between the reduced principal subtree of an LGT network and its reduced secondary subtrees. However, since these trees need not be binary, we slightly generalize the rSPR operations defined in [30] to allow for the pruned subtree to be regrafted not only to an arc but also to a node.

More precisely, we define an rSPR operation of a tree T as the following procedure:

1. Choose an arc $e=(u,v)$ of *T*.
2. Remove *e* from *T*.
3. Choose a node *w* that is not a descendant of *v*.
4. If *w* is an internal node other than *u*, then apply either (a) or (b) below. If *w* is a leaf or $w=u$, apply (b).
  - Add an arc (*w*, *v*).
  - Add a new node $\widetilde{w}$ and new arcs $(\widetilde{w},v)$ and $(\widetilde{w},w)$. If *w* was not the root of *T* and $w{^{\prime }}$ was its parent, then remove the arc $(w{^{\prime }},w)$ and add a new arc $(w{^{\prime }}, \widetilde{w})$. If *w* was the root, then $\widetilde{w}$ becomes the root of the resulting tree.
5. Suppress *u* if it has become elementary.

We shall denote such an rSPR operation by $v \mathop {\longleftarrow }\limits ^{{node}}w$ (a *node rSPR operation*) if step (4a) is applied, and $v \mathop {\longleftarrow }\limits ^{{arc}}w$ (an *arc rSPR operation*) if step (4b) is applied; cf. Fig. 5. When it is not necessary to specify whether it is a node or an arc rSPR operation, we shall denote it by $v \mathop {\longleftarrow }\limits ^{{spr}}w$.

Given any pair of phylogenetic trees on the same set of labels, their *rSPR distance*$d_{rSPR}(T,T{^{\prime }})$ is the least number of rSPR operations that transform one into the other (cf. [30] in the binary case). In particular, since a reduced secondary subtree $\widetilde{T}_e(N)$ of an LGT network is obtained from its reduced principal subtree $\widetilde{T}_0(N)$ by means of an rSPR operation, we have that $d_{rSPR}(\widetilde{T}_0(N),\widetilde{T}_e(N))\le 1$, and $d_{rSPR}(\widetilde{T}_0(N),\widetilde{T}_e(N))= 1$ if, and only if, $\widetilde{T}_0(N)\ne \widetilde{T}_e(N)$.

An *isomorphism* of LGT networks is an isomorphism of *S*-rDAG that preserves and reflects the partitions of the sets of arcs into principal and secondary. More formally, given two LGT networks $N=(V,E)$ and $N{^{\prime }}=(V{^{\prime }},E{^{\prime }})$, an isomorphism from *N* to $N{^{\prime }}$ is a bijection $\phi : V\rightarrow V{^{\prime }}$ such that:

- (*u*, *v*) is a principal arc in *N* if, and only if, $(\phi (u),\phi (v))$ is a principal arc in $N{^{\prime }}$;
- (*u*, *v*) is a secondary arc in *N* if, and only if, $(\phi (u),\phi (v))$ is a secondary arc in $N{^{\prime }}$;
- $u\in V$ is a leaf labelled with $s\in S$ if, and only if, $\phi (u)$ is a leaf labelled with *s*.

The isomorphism of LGT networks can be easily checked in linear time in their sizes. Indeed, two LGT networks *N* and $N{^{\prime }}$ are isomorphic if, and only if, $T_0(N)= T_0(N{^{\prime }})$—which can be checked in linear time in the number of principal arcs of the networks—and this isomorphism preserves and reflects the sets of secondary arcs.

As we do with *S*-rDAG in general, we shall usually say that two LGT networks are *equal* when they are actually isomorphic.

## A reconstruction problem for a restricted class of LGT networks

Let us consider the problem of reconstructing an LGT network from its reduced principal subtree $T_0$ and its set of reduced secondary subtrees $T_1,\ldots ,T_k$. We shall take into account only the case when $T_1,\ldots ,T_k$ are pairwise different, because if $T_i=T_j$, they can be defined by the same secondary arc. Moreover, we shall restrict ourselves to the case when $T_0\ne T_i$ for every $i=1,\ldots ,k$, because when a reduced secondary subtree is equal to the reduced principal subtree, it only means that we are not able to “distinguish” the secondary line of evolution from the principal one. This leads us to the following general problem:

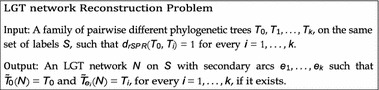



**Fig. 6:**
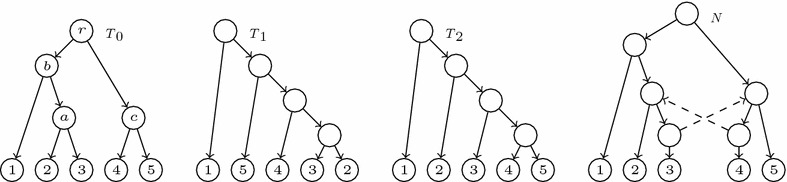
Any “LGT network” with reduced principal subtree $T_0$ and reduced secondary subtrees $T_1,T_2$ would contain a cycle

Of course, this problem may have no solution for certain input trees. Consider, for instance, the trees $T_0,T_1,T_2$ depicted in Fig. 6. A simple inspection shows that if there exists an LGT network *N* with reduced principal subtree $T_0$ and two secondary arcs $e_1,e_2$ such that $\widetilde{T}_{e_1}(N)= T_1$ and $\widetilde{T}_{e_2}(N)= T_2$, then $e_1$ must go from an elementary node added in the arc ending in 4 to *a* (or to an elementary node added in the arc ending in *a*), and $e_2$ must go from an elementary node added in the arc ending in 3 to *c* (or to an elementary node added in the arc ending in *c*). But then, the resulting directed graph contains a cycle: see, for instance, the graph *N* in Fig. 6.

**Fig. 7:**
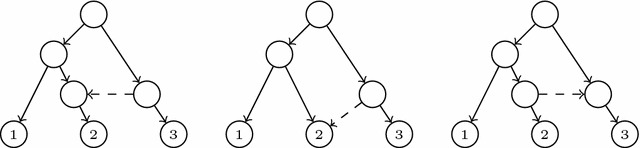
Three LGT networks with the same reduced principal and secondary subtrees

**Fig. 8:**
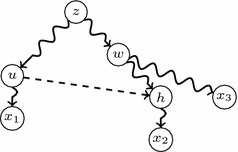
The structure of *N* involving *e* in the proof of Proposition 2

**Fig. 9:**
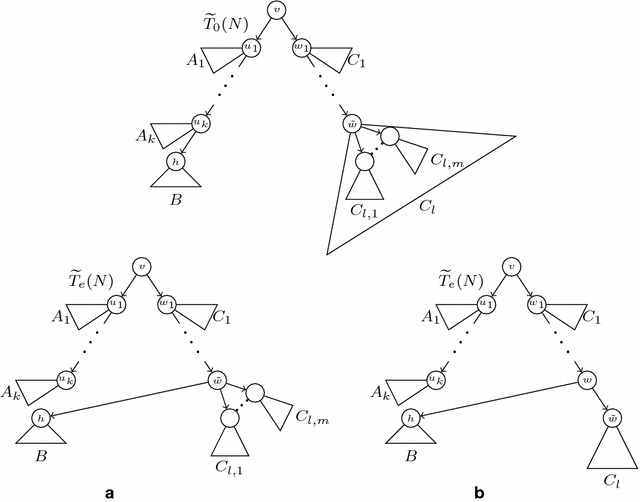
The local structure of $\widetilde{T}_0(N)$ and $\widetilde{T}_e(N)$ around a secondary arc $e=(w,h)$, when *w* is not principally elementary (**a**) and when it is principally elementary (**b**)

**Fig. 10:**
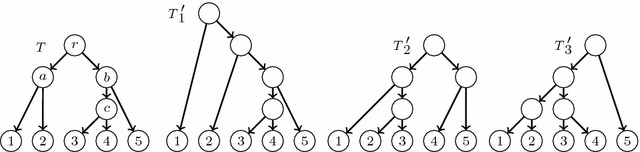
The phylogenetic trees used as input in Example 1

On the other hand, as it was already hinted in the discussion above, if the LGT network reconstruction problem has a solution for a specific input, it need not be unique: see, for instance, Fig. 7. And, as we mentioned at the beginning of this section, there may be repetitions in the family of reduced principal and secondary subtrees of a general LGT network, and therefore not every LGT network can be obtained as an output of this problem.

This motivates us to restrict ourselves to a class of LGT networks satisfying a set of conditions that guarantee, on the one hand, that their reduced principal and secondary subtrees are pairwise different and, on the other hand, the uniqueness of the restricted LGT network with given reduced principal and secondary subtrees, if some exists.

### **Definition 2**

An LGT network is *restricted* when it satisfies the following properties:

- No principal child of a principally elementary node is principally elementary.
- The target of a secondary arc is never principally elementary.
- If (*u*, *h*) is a secondary arc, then there exists no principal path $u\!\rightsquigarrow \!{}h$.
- If (*u*, *h*) is a secondary arc and $z=LCA_{T_0(N)}(u,h)$, then the principal path $z\!\rightsquigarrow \!{}h$ contains some non principally elementary intermediate node.

Conditions (a) and (b) are necessary to guarantee the uniqueness of the solutions:

- Let *N* be an LGT network with a principal arc $(u,u{^{\prime }})$ with both $u,u{^{\prime }}$ principally elementary: then (since *N* cannot contain elementary nodes) both $u,u{^{\prime }}$ must be sources of secondary arcs, say $e=(u,h)$ and $e{^{\prime }}=(u{^{\prime }},h{^{\prime }})$. If $h=h{^{\prime }}$, these arcs define the same reduced secondary subtree. If $h\ne h{^{\prime }}$, then, if we replace *e* and $e{^{\prime }}$ by $\bar{e}=(u,h{^{\prime }})$ and $\bar{e}{^{\prime }}=(u{^{\prime }},h)$, we obtain a new LGT network with the same reduced principal and secondary subtrees as *N*.
- Let *N* be an LGT network with a secondary arc $e=(u,h)$ with *h* principally elementary, and let $h{^{\prime }}$ be the principal child of *h*. We shall assume that *N* does not contain the secondary arc $e{^{\prime }}=(u,h{^{\prime }})$, because otherwise $\widetilde{T}_e(N)=\widetilde{T}_{e{^{\prime }}}(N)$. Then, if we replace the secondary arc (*u*, *h*) by a secondary arc $(u,h{^{\prime }})$, we obtain a new LGT network with the same reduced principal and secondary subtrees as *N*.

As far as the other two conditions go, (c) prevents the existence of a lateral gene transfer from a species to a principal descendant of it, and condition (d) prevents the existence of a lateral gene transfer from a species to a species represented by an ancestor of it in the reduced principal subtree.

Except for (c), which is shared by both definitions, the conditions that define our restricted LGT networks are transversal to those defining species graphs.

We shall prove now that the reduced principal and secondary subtrees of a restricted LGT network form a family of pairwise different phylogenetic trees.

### **Proposition 2**

*IfN is a restricted LGT network ande* i*s a secondary arc in it, then *$\widetilde{T}_0(N)\ne \widetilde{T}_e(N)$.

### *Proof*

Let $e=(u,h)\in E_s$; to simplify the notations, we shall denote $T_0(N)$ and $T_e(N)$ by $T_0$ and $T_e$, respectively. We shall prove that these trees define different sets of triples; by Proposition 1, this will imply that $\widetilde{T}_0 \ne \widetilde{T}_e$.

By condition (c) in Definition 2, there exists no principal path connecting *u* and *h*, and therefore $C_{T_0}(h)\cap C_{T_0}(u)=\emptyset$. Let $x_1\in C_{T_0}(u)$ and $x_2\in C_{T_0}(h)$. On the other hand, if $z=LCA_{T_0}(u,h)$, condition (d) in Definition 2 implies that the principal path $z\!\rightsquigarrow \!{}h$ contains some intermediate node *w* with a principal child $w_1$ outside this path; let $x_3\in C_{T_0}(w_1)$ (see Fig. 8). It is straightforward to check now that $T_0$ defines the triple $((x_2,x_3),x_1)$ and $T_e$ defines the triple $((x_1,x_2),x_3)$. Therefore, $\Gamma (T_0)\ne \Gamma (T_e)$, as we claimed. $\square$

### **Proposition 3**

*If N is a restricted LGT network and *$e,e{^{\prime }}$* are two different secondary arcs in it, then*$\widetilde{T}_e(N)\ne \widetilde{T}_{e{^{\prime }}}(N)$.

The proof of this proposition is similar to that of Proposition 2, but much longer because we must distinguish many cases, depending on the relative positions of the source and the target nodes of *e* and $e{^{\prime }}$ in $T_0(N)$. Therefore, and in order not to lose the thread of the paper, we postpone it until the Additional file 1: Appendix.

The problem we are actually going to solve in this section is, then, the following special case of the LGT Network Reconstruction Problem:

Our next goal is now to establish a set of necessary and sufficient conditions for the existence of a restricted LGT network *N* with a given principal subtree *T* and a given secondary subtree $T{^{\prime }}$. First, we give these conditions in terms of rSPR operations. Next, we translate the resulting conditions in terms of triples and clusters.

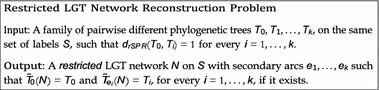



### **Proposition 4**

*Let*$T,T{^{\prime }}$* be two phylogenetic trees on the same set of labels. There exists a restricted LGT networkN with a secondary arce such that *$T = \widetilde{T}_0(N)$* and *$T{^{\prime }} = \widetilde{T}_e(N)$* if, and only if:*

1. $$d_{rSPR}(T,T{^{\prime }})=1$$,* and*
2. If $h \mathop {\longleftarrow }\limits ^{{spr}}w$* is an rSPR operation that produces*$T{^{\prime }}$* fromT*,* then, in T*, *w is neither an ancestor ofh nor a descendant of the parent of h*.

### *Proof*

As far as the necessity of conditions (1) and (2) goes, recall from § that, if *N* is an LGT network and $e=(u,h)$ a secondary arc in it, then $\widetilde{T}_e(N)$ is obtained from $\widetilde{T}_0(N)$ by means of either a node rSPR operation $h \mathop {\longleftarrow }\limits ^{{node}}u$, when *u* is not principally elementary in *N*, or an arc rSPR operation $h \mathop {\longleftarrow }\limits ^{{arc}}u^*$, with $u^*$ the only principal child of *u* in *N*, when it is principally elementary. Since, moreover, $\widetilde{T}_e(N) \ne \widetilde{T}_0(N)$ by Proposition 2, this entails that $d_{rSPR}(T,T{^{\prime }})=1$. On the other hand, *u* (or $u^*$, in the second case) can be neither a principal ancestor of *h*, because of condition (c) in Definition 2, nor a proper principal descendant of the parent *v* of *h* in $\widetilde{T}_0(N)$, because this would imply that $v=LCA_{T_0}(u,h)$, against condition (d) in Definition 2.

Let us prove now the sufficiency of conditions (1) and (2). If $T{^{\prime }}$ is obtained from *T* by means of a node rSPR operation $h \mathop {\longleftarrow }\limits ^{{node}}w$, let *N* be the LGT network obtained by adding to *T* the secondary arc (*w*, *h*). If $T{^{\prime }}$ is obtained by means of an arc rSPR operation $h \mathop {\longleftarrow }\limits ^{{arc}}w$, then, since *h* is not a descendant of *w* in *T*, the latter cannot be the root; in this case, if *v* is its parent in *T*, split the arc (*v*, *w*) by adding an intermediate node *u* in it, and add a secondary arc $e=(u,h)$; let *N* be the resulting LGT network.

In both cases, it is clear by construction that $\widetilde{T}_0(N) = T$ and $\widetilde{T}_e(N) = T{^{\prime }}$. Moreover, *N* clearly satisfies condition (a) (because *N* has at most one principally elementary node), (b) (because *h* is not elementary in *T*), (c) (because *h* is not a descendant of *w* in *T*), and (d) (because, since *w* is not a descendant in *T* of the parent $h_0$ of *h*, the path $LCA_T(w,h)\!\rightsquigarrow \!{}h$ in $T_0(N)$ contains $h_0$ as intermediate node, and it is not elementary in *T*) in the definition of restricted LGT network. $\square$

We rewrite now the characterization provided by the previous proposition in terms of triples (Proposition 5) and clusters (Proposition 6).

We say that two trees $T, T{^{\prime }}$ on the same set of labels *S* and given by their respective set of triples $\{T_{x,y,z}\mid \{x,y,z\}\subseteq S\}$ and $\{T{^{\prime }}_{x,y,z}\mid \{x,y,z\}\subseteq S\}$ satisfy the *principal-secondary condition on triples* if there exist $k,l,m\ge 1$ and a family of non-empty, pairwise disjoint subsets of *S*

$$\begin{aligned} A_1,\ldots , A_k, B,C_1,\ldots , C_{l-1},C_{l,1},\ldots ,C_{l,m} \end{aligned}$$

(and to ease notations, let $C_l=\bigcup \nolimits _{i=1}^m C_{l,i}$) such that for every $x,y,z\in S$:

1. If $x\in \bigcup \nolimits _{i=1}^k A_i$, $y\in B$, and $z\in \bigcup \nolimits _{i=1}^l C_i$, then $T_{x,y,z}=((x,y),z)$ and $T{^{\prime }}_{x,y,z}=((y,z),x)$.
2. If $x\in B$, $y\in A_j$ and $z\in A_i$, for some $1\le i<j\le k$, then $T_{x,y,z}=((x,y),z)$ and $T{^{\prime }}_{x,y,z}=((y,z),x)$.
3. If $x\in C_i$, $y\in C_j$ and $z\in B$, for some $1\le i<j\le l$, then $T_{x,y,z}=((x,y),z)$ and $T{^{\prime }}_{x,y,z}=((y,z),x)$.
4. If $x\in C_{l,i}$, $y\in C_{l,j}$ and $z\in B$, for some $1\le i<j\le m$, then $T_{x,y,z}=((x,y),z)$ and $T{^{\prime }}_{x,y,z}=(x,y,z)$.
5. If *x*, *y*, *z* do not satisfy any of the previous conditions, then $T_{x,y,z}=T{^{\prime }}_{x,y,z}$.

### **Proposition 5**

*Let *$T,T{^{\prime }}$* be two phylogenetic trees on the same set of labels. There exists a restricted LGT network N with a secondary arc e* such that $T = \widetilde{T}_0(N)$* and *$T{^{\prime }} = \widetilde{T}_e(N)$* if, and only if, they satisfy the principal-secondary condition on triples*.

### *Proof*

As far as the “only if” implication goes, assume that $e=(w,h)$ and let $v=LCA_{T_0(N)}(w,h)=LCA_{\widetilde{T}_0(N)}(w,h)$. Let $\tilde{w}\in \widetilde{T}_0(N)$ be the first non principally elementary principal descendant of *w*: that is, $\tilde{w}=w$ if *w* is not principally elementary, and its principal child otherwise. Now:

- Let $v\rightarrow u_{1}\rightarrow \cdots \rightarrow u_k\rightarrow h$ be the path $v\!\rightsquigarrow \!{}h$ in $\widetilde{T}_0(N)$ [where $k\ge 1$ by condition (d) in Definition 2];
- Let $v\rightarrow w_1\rightarrow \cdots \rightarrow w_{l-1}\rightarrow w_l=\tilde{w}$ be the path $v\!\rightsquigarrow \!{}\tilde{w}$ in $\widetilde{T}_0(N)$ [where $l\ge 1$ because condition (c) in Definition 2 implies that $w\ne v$];
- For every $i=1,\ldots , k-1$, let $A_i=C_{T_0(N)}(u_i){\setminus } C_{T_0(N)}(u_{i+1})$;
- Let $$A_k=C_{T_0(N)}(u_k){\setminus } C_{T_0(N)}(h)$$;
- Let $$B=C_{T_0(N)}(h)$$;
- For every $i=1,\ldots , l-1$, let $C_i=C_{T_0(N)}(w_i){\setminus } C_{T_0(N)}(w_{i+1})$;
- If $\tilde{w}=w$, let $x_1,\ldots ,x_m$ be its children in $\widetilde{T}_0(N)$, and let $C_{l,i}=C_{T_0(N)}(x_i)$, for $i=1,\ldots ,m$; if *w* is principally elementary in *N*, let $C_l=C_{l,1}=C_{\widetilde{T}_0(N)}(\tilde{w})=C_{T_0(N)}({w})$.

(Cf. Fig. 9). It is straightforward to check that the triples defined by $T_0(N)$ and $T_e(N)$ are the same except for those in the statement.

Let us consider now the “if” implication. In order not to overload the text, we shall outline here the proof, and fill in the details in a series of Claims proved in the Additional file 1: Appendix.

Assuming that the symmetric difference $\Gamma (T)\bigtriangleup \Gamma (T{^{\prime }})$ consists of those triples described in the statement, we have that *B* is a cluster of both *T* and $T{^{\prime }}$ (this is Claim 1 in the Appendix, where it is proved). Since every triple in $\Gamma (T)\bigtriangleup \Gamma (T{^{\prime }})$ involves one, and only one, leaf in *B*, it is clear that $\Gamma (T|_B)=\Gamma (T{^{\prime }}|_{B})$ and $\Gamma (T|_{S{\setminus } B})=\Gamma (T{^{\prime }}|_{S{\setminus } B})$ and hence $T|_B= T{^{\prime }}|_B$ and $T|_{S{\setminus } B}= T{^{\prime }}|_ {S{\setminus } B}$. So, $T|_B$ and $T|_{S{\setminus } B}$ form a maximum-agreement forest for *T* and $T{^{\prime }}$ in the sense of [31], which implies that $d_{rSPR}(T,T{^{\prime }})=1$ [30, Theorem 2.1]. Then, the rSPR operation that transforms *T* into $T{^{\prime }}$ must have the form $h \mathop {\longleftarrow }\limits ^{{spr}}x$, with *h* the root of $T|_B$, that is, the node in *T* with $C_T(h)=B$. In order to prove that this rSPR operation satisfies condition (2) in Proposition 4, we must identify the node *x* and the type of rSPR operation. To do that, we use that each $C_{l,i}$ is a cluster in *T* and $T{^{\prime }}$ (cf. Claim 2 in the Appendix) and that $B\cup C_{l}$ is a cluster in $T{^{\prime }}$ but not in *T* (cf. Claim 3). Then:

- If $m=1$, so that $C_l=C_{l,1}\in C(T)\cap C(T{^{\prime }})$, this entails that the nodes with clusters *B* and $C_{l}$ are sibling in $T{^{\prime }}$ but not in *T*, and therefore that *x* is the node in *T* with cluster $C_l$ and that the rSPR operation is of type arc.
- If $m>1$, since $C_{l}$ is a cluster in *T* but not in $T{^{\prime }}$ (this is Claim 4 in the Appendix) and $B\cup C_{l,i_1}\cup \cdots \cup C_{l,i_k}\notin C(T{^{\prime }})$ for every $\emptyset \ne \{i_1,\ldots ,i_k\}\subsetneq \{1,\ldots ,m\}$ (cf. Claim 5), we have that the nodes with clusters $B,C_{l,1},\ldots ,C_{l,m}$ are sibling in $T{^{\prime }}$ but not in *T*, and therefore that *x* is the node in *T* with cluster $C_l$ and that the rSPR operation is of type node.

In both cases, it is easy to see that *x* is not connected in *T* with *h* (because $B\cap C_l=\emptyset$) and that $LCA_T(x,h)$ is not the parent of *h* (because if $a\in A_1$, $b\in B$ and $c\in C_l$, then $((a,b),c)\in \Gamma (T)$). $\square$

### **Corollary 1**

*LetN and*$N{^{\prime }}$* be two restricted LGT networks on the same set of labels S*,* each with a single secondary arc: say*, *e and *$e{^{\prime }}$,* respectively. If *$\widetilde{T}_0(N)= \widetilde{T}_0(N{^{\prime }})$* and*$\widetilde{T}_e(N)= \widetilde{T}_{e{^{\prime }}}(N{^{\prime }})$,* then *$N=N{^{\prime }}$.

### *Proof*

Let us denote $\widetilde{T}_0(N)= \widetilde{T}_0(N{^{\prime }})$ simply by *T*. Since *N* and $N{^{\prime }}$ are restricted LGT networks, the proof of the last proposition shows that if $\widetilde{T}_e(N)= \widetilde{T}_{e{^{\prime }}}(N{^{\prime }})$, then *e* and $e{^{\prime }}$ must have the same source and target nodes: with the notations therein, their target node is the node in *T* with cluster *B*, and their source node is either a principally elementary node added in the arc ending in the node in *T* with cluster $C_l$ (if $m=1$) or the node in *T* with cluster $C_l$ (if $m>1$). Therefore, $N=N{^{\prime }}$. $\square$

Notice that the naïve implementation of the procedure given by Proposition 5, that computes and writes all the $O(n^3)$ triples defined by *T* and $T{^{\prime }}$ and then checks whether the symmetric difference of the corresponding sets of triples has the form described therein, takes at least $O(n^4)$ time. Although this cost can possibly be reduced by using the strategy in [32], we found it simpler to translate this condition on triples into an equivalent condition on clusters that is faster to check. To this end we first give a set of conditions written in terms of clusters of trees and its structure as a partial ordered set, where we consider the natural ordering given by inclusion of sets. In the context of posets, a *segment* is a chain such that every element in the poset lying between the ends of the chain also belongs to the chain.

We say that two trees $T,T{^{\prime }}$ on the same set of labels *S* and given by their respective set of clusters *C*(*T*) and $C(T{^{\prime }})$ satisfy the *pricipal-secondary condition on clusters* if:

- The symmetric difference of the clusters of *T* and $T{^{\prime }}$ can be written as follows: There exist $k,l\ge 1$ such that:
  $C(T) {\setminus } C(T{^{\prime }})$ consists (at most) of two maximal disjoint segments in *C*(*T*)
    $$\begin{aligned} U_k \subsetneq \cdots \subsetneq U_1,\quad W_{l_0} \subsetneq \cdots \subsetneq W_1, \end{aligned}$$
    with $$l-1\le l_0\le l$$.
  $C(T{^{\prime }}) {\setminus } C(T)$ consists (at most) of two maximal disjoint segments in $C(T{^{\prime }})$
    $$\begin{aligned} U_{k_0}{^{\prime }} \subsetneq \cdots \subsetneq U{^{\prime }}_1,\quad W{^{\prime }}_{l} \subsetneq \cdots \subsetneq W{^{\prime }}_1, \end{aligned}$$
    with $$k-1\le k_0\le k$$.
  If $l=1$ and $l_0=l-1$, (respectively, if $k=1$ and $k_0=k-1$), the chain $W_{l_0} \subsetneq \cdots \subsetneq W_1$ (respectively, $U_{k_0}{^{\prime }} \subsetneq \cdots \subsetneq U{^{\prime }}_1$) does not exist, and then $C(T) {\setminus } C(T{^{\prime }})$ (respectively, $C(T{^{\prime }}) {\setminus } C(T)$) consists only of the other segment.
  If $C(T) {\setminus } C(T{^{\prime }})$ (respectively, $C(T{^{\prime }}) {\setminus } C(T)$) consists of two maximal disjoint segments of clusters, then $U_1\cap W_1=\emptyset$ (respectively, $U_1{^{\prime }}\cap W_1{^{\prime }}=\emptyset$).
- The minimal elements in the chains above satisfy that $U_k\cap W_l{^{\prime }}\in C(T)\cap C(T{^{\prime }})$. Let *B* denote this cluster.
- The difference between the first element in the first segment and the common cluster *B*, say $A_k=U_k{\setminus } B$ satisfies:
  $$A_k\in C(T{^{\prime }})$$;
  if $$k_0=k-1$$, then $$A_k\in C(T)$$;
  if $$k_0=k$$, then $$U{^{\prime }}_k=A_k\notin C(T)$$.
- Analogously, the difference between the first element in the last segment and the common cluster *B*, say $C_l= W{^{\prime }}_{l}{\setminus } B$ satisfies:
  $$C_l\in C(T)$$;
  if $$l_0=l-1$$, then $$C_l\in C(T{^{\prime }})$$;
  if $$l_0=l$$, then $$W_l=C_l\notin C(T{^{\prime }})$$.
- If $k>1$, the differences between consecutive sets in the segments above satisfy:
  $$A_k\subsetneq U_{k-1}{^{\prime }}$$;
  Setting (even when $k_0=k-1$) $U_{k}{^{\prime }}=A_k$, we have that $U_i{\setminus } U_{i+1}=U_i{^{\prime }}{\setminus } U_{i+1}{^{\prime }}$ for every $i=1,\ldots , k-1$.
- And analogously, if $l>1$, then:
  $$C_l\subsetneq W_{l-1}$$;
  Setting (even when $l_0=l-1$) $W_{l}=C_l$, we have that $W_i{\setminus } W_{i+1}=W_i{^{\prime }}{\setminus } W_{i+1}{^{\prime }}$ for every $i=1,\ldots , l-1$.

### **Proposition 6**

*Let *$T,T{^{\prime }}$* be two different phylogenetic trees on the same set of labels. There exists a restricted LGT networkN with a secondary arce such that*$T = \widetilde{T}_0(N)$* and*$T{^{\prime }} = \widetilde{T}_e(N)$* if, and only if they satisfy the principal-secondary condition on clusters*.

The principal-secondary condition on clusters can be checked in $O(n^2)$ time. Indeed, conditions (b) to (f) can be checked in linear time, since they only involve testing if certain sets are clusters of the trees or subsets of some specific sets of leaves. As for condition (a), one only needs to compute all the clusters of both trees, which can be done in $O(n^2)$ time, and then computing the symmetric difference of those sets and arranging this symmetric difference in chains, which can be done in linear time in the size of the clusters.

Proposition 6 allows us to detect easily the secondary arc that must be added to *T* in order to obtain a network that has $T{^{\prime }}$ as the corresponding reduced secondary tree, when it exists, by means of the following algorithm:

It turns out that $N(T,T{^{\prime }})$ is contained in every restricted LGT network with reduced principal subtree *T* and having $T{^{\prime }}$ as a reduced secondary subtree.

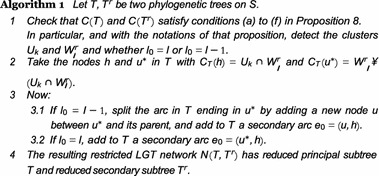



### **Proposition 7**

*Let N be a restricted LGT network such that *$\widetilde{T}_0(N)= T$* and *$\widetilde{T}_e(N)= T{^{\prime }}$,* for some secondary arce*. Let $N{^{\prime }}$* be the LGT network obtained by removing fromN all secondary arcs except e and then suppressing elementary nodes. Then, *$N{^{\prime }}=N(T,T{^{\prime }})$.

### *Proof*

In this situation, $N{^{\prime }}$ is also a restricted LGT network with $\widetilde{T}_0(N{^{\prime }})= T$ and $\widetilde{T}_e(N{^{\prime }})= T{^{\prime }}$, and then Corollary 1 applies.$\square$

Now we are able to solve the Restricted LGT Network Reconstruction problem:

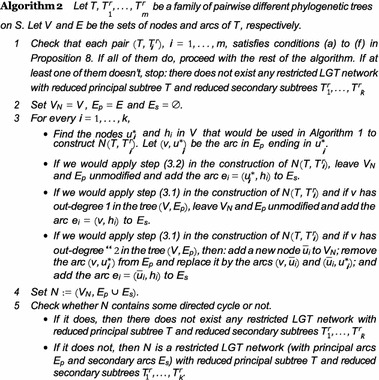



### **Proposition 8**

*Let *$T,T{^{\prime }}_1,\ldots ,T_k{^{\prime }}$* be a family of pairwise different phylogenetic trees onS such that each pair*$(T,T_i{^{\prime }})$, $i=1,\ldots ,k$,* satisfies conditions (a) to (f) in Proposition *6.* If there exists some restricted LGT network*$\bar{N}$* with reduced principal subtreeT and reduced secondary subtrees*$T_1{^{\prime }},\ldots ,T_k{^{\prime }}$,* then the graphN defined in step 4 of Algorithm 2 applied to*$T,T{^{\prime }}_1,\ldots ,T_k{^{\prime }}$* is equal to *$\bar{N}$* (up to isomorphisms of LGT networks)*.

### *Proof*

Let $\bar{N}$ be a restricted LGT network with $\widetilde{T}_0(\bar{N})=T$ and reduced secondary subtrees $T{^{\prime }}_1,\ldots ,T_k{^{\prime }}$. Without any loss of generality, we rename these reduced secondary subtrees as $T{^{\prime }}_{1,1},\ldots ,T{^{\prime }}_{1,k_1},T{^{\prime }}_{2,1},\ldots ,T_{l,k_l}{^{\prime }}$ ($k_1+\cdots +k_l=k$) in such a way that, for every $i=1,\ldots , l$, the secondary arcs $\bar{e}_{i,1},\ldots ,\bar{e}_{i,k_i}$ producing the reduced secondary subtrees $T{^{\prime }}_{i,1},\ldots ,T{^{\prime }}_{i,k_i}$ have the same origin $u_i$, and $u_i\ne u_j$ if $i\ne j$. For every $i=1,\ldots , l$, let $u_i^*$ be equal to $u_i$ if this node is not principally elementary, and to the principal child of $u_i$ in $\bar{N}$ if it is principally elementary; in both cases, $u_i^*$ is a node in *T*. Finally, for every $i=1,\ldots , l$ and $j=1,\ldots , k_i$, let $h_{i,j}$ be the target of $\bar{e}_{i,j}$, which is also a node in *T*.

We know from Proposition 6 (and its proof) that the clusters of each $u_i^*$ and each $h_{i,j}$ and the equality, or not, between $u_i$ and $u_i^*$ are uniquely determined by the pair $(T,T_i{^{\prime }})$. Indeed, in each case the clusters of the aforementioned nodes are found in the proof of Proposition 8, and the statement of this proposition shows how these clusters are determined by *T* and $T{^{\prime }}_i$. Then, we can understand that Algorithm 2 first splits the arc in *T* ending in each $u_i^*$ for which $u_i\ne u_i^*$ into two arcs connected by a new elementary node $\bar{u}_i$ and next, for every $i=1,\ldots ,l$ and $j=1,\ldots ,k_i$, adds to the resulting *S*-tree a secondary arc from $\bar{u}_i$ or from $u_i^*$ to $h_{i,j}$. It is clear then that the resulting graph *N* is isomorphic to $\bar{N}$ by means of an isomorphism that preserves labels, principal arcs and secondary arcs. $\square$

This proposition entails, on the one hand, that if there exists some restricted LGT network with reduced principal subtree *T* and reduced secondary subtrees $T_1{^{\prime }},\ldots ,T_k{^{\prime }}$, then it is unique (up to isomorphisms), and, on the other hand, that Algorithm 2 is correct (and also independent of the ordering of the trees $T_1{^{\prime }},\ldots ,T_k{^{\prime }}$), in the sense that such a restricted LGT network exists if, and only if, the algorithm finds it: notice that if the algorithm detects a cycle in step 5, then this proposition implies that no restricted LGT network can have *T* and $T{^{\prime }}_1,\ldots ,T_k{^{\prime }}$ as reduced principal and reduced secondary subtrees. Another consequence is the stability of the network reconstructed: If some new tree is added to the input of the algorithm, then a new secondary arc is added to the network, without altering the other secondary arcs (notice, however, that this last secondary arc could create a cycle in the network and hence the problem would have no solution).

The following examples show two simple applications of Algorithm 2.

### *Example 1*

Consider the trees depicted in Fig. 10.

- $C(T){\setminus } C(T_1{^{\prime }})=\big \{\{1,2\}\big \}$ and $C(T_1{^{\prime }}){\setminus } C(T)=\big \{\{2,3,4,5\}\big \}$. Then, with the notations of Algorithm 2, $k=l=1$, $k_0=l_0=0$, $U_k=\{1,2\}$, $W_l{^{\prime }}=\{2,3,4,5\}$, $B=\{2\}$, $C_l=\{3,4,5\}$, $u_1^*=b$, and $h_1=2$. So, we add a new principally elementary node in the middle of the arc (*r*, *b*) and a secondary arc $e_1$ from it to 2.
- $$C(T){\setminus } C(T_2{^{\prime }})=\big \{\{1,2\},\{3,4\},\{3,4,5\}\big \}$$ and $$C(T_2{^{\prime }}){\setminus } C(T)=\big \{\{2,3\},\{1,2,3\},$$$$\{ 4,5\}\big \}$$. Then, $$k=l=2$$, $$k_0=l_0=1$$, $$U_k=\{3,4\}$$, $$W_l{^{\prime }}=\{2,3\}$$, $$B=\{3\}$$, $$C_l=\{2\}$$, $$u_2^*=2$$ and $$h=3$$. So, we add a new principally elementary node in the middle of the arc (*a*, 2) and a secondary arc $$e_2$$ from it to 3.
- $C(T){\setminus } C(T_3{^{\prime }})=\big \{\{3,4,5\}\big \}$ and $C(T_3{^{\prime }}){\setminus } C(T)=\big \{\{1,2,3,4\}\big \}$. Then, $k=l=1$, $k_0=l_0=0$, $U_k=\{3,4,5\}$, $W_l{^{\prime }}=\{1,2,3,4\}$, $B=\{3,4\}$, $C_l=\{1,2\}$, $u_3^*=a$ and $h_3=c$. So, we add a new principally elementary node in the middle of the arc (*r*, *a*) and a secondary arc $e_3$ from it to *c*.

**Fig. 11:**
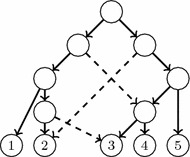
The graph obtained as output when applying Algorithm 2 to the trees $T,T_1{^{\prime }},T_2{^{\prime }},T_3{^{\prime }}$ in Fig. 10

We obtain the directed graph depicted in Fig. 11, which is acyclic and therefore a restricted LGT network with reduced principal subtree *T* and reduced secondary subtrees $T_1{^{\prime }},T_2{^{\prime }},T_3{^{\prime }}$.

**Fig. 12:**
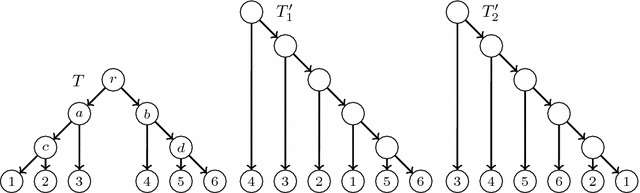
The phylogenetic trees used as input in Example 2

**Fig. 13:**
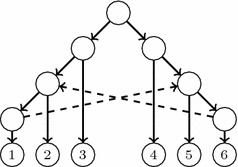
The graph obtained as output when applying Algorithm 2 to the trees $T,T_1{^{\prime }},T_2{^{\prime }}$ in Fig. 12

**Fig. 14:**
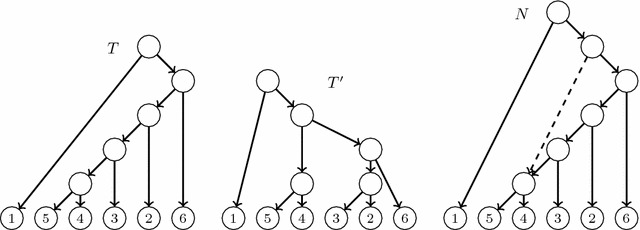
The phylogenetic trees used as input in Example 3, and an LGT network that has them as reduced principal and secondary subtrees, respectively

### *Example 2*

Consider the trees depicted in Fig. 12.

- $$C(T){\setminus } C(T_1{^{\prime }})=\big \{\{1,2\},\{1,2,3\},\{4,5,6\}\big \}$$ and $$C(T_1{^{\prime }}){\setminus } C(T)=\big \{\{1,5,6\},$$$$\{1,2,5,6\},\{1,2,3,5,6\}\big \}$$. Then, $$k=1$$, $$l=3$$, $$k_0=0$$, $$l_0=2$$, $$U_k=\{4,5,6\}$$, $$W_l{^{\prime }}=\{1,5,6\}$$, $$B=\{5,6\}$$, $$C_l=\{1\}$$, $$u_1^*=1$$ and $$h_1=d$$. So, we add a new principally elementary node in the middle of the arc (*c*, 1) and a secondary arc $$e_1$$ from it to *d*.
- $$C(T){\setminus } C(T_2{^{\prime }})=\big \{\{1,2,3\},\{5,6\},\{4,5,6\}\big \}$$ and $$C(T_2{^{\prime }}){\setminus } C(T)=\big \{\{1,2,6\},$$$$\{1,2,5,6\},\{ 1,2,4,5,6\}\big \}$$. Then, $$k=1$$, $$l=3$$, $$k_0=0$$, $$l_0=2$$, $$U_k=\{1,2,3\}$$, $$W_l{^{\prime }}=\{1,2,6\}$$, $$B=\{1,2\}$$, $$C_l=\{6\}$$, $$u_2^*=6$$ and $$h_2=c$$. So, we add a new principally elementary node in the middle of the arc (*d*, 6) and a secondary arc $$e_2$$ from it to *c*.

We obtain the directed graph depicted in Fig. 13, which contains a directed cycle. Therefore, there does not exist any restricted LGT network with *T* as reduced principal subtree and $T_1{^{\prime }},T_2{^{\prime }}$ as reduced secondary subtrees.

Of course, it is possible that, on a given input, the LGT network Reconstruction Problem has a solution and the Restricted LGT network Reconstruction Problem does not, as the following example shows.

### *Example 3*

Consider the trees $T,T{^{\prime }}_1$ depicted in Fig. 14.

Then, $C(T){\setminus } C(T{^{\prime }}_1)=\big \{\{3,4,5\},\{2,3,4,5\}\big \}$ and $C(T{^{\prime }}_1){\setminus } C(T)=\big \{\{2,3\},\{2,3,6\}\big \}$, and therefore these trees do not satisfy conditions (a) to (f) in Proposition 6: from $C(T){\setminus } C(T{^{\prime }}_1)$ we have that $k=2$, and from $C(T{^{\prime }}_1){\setminus } C(T)$ that $l=2$, but then both differences should consist of a pair of segments, instead of a single segment. This means that there does not exist any restricted LGT network with reduced principal subtree *T* and reduced secondary subtree $T{^{\prime }}_1$. But there actually exists an LGT network with reduced principal subtree *T* and reduced secondary subtree $T{^{\prime }}_1$: the network *N* depicted in the same figure, which is not restricted.

## An application

In order to test the models and algorithms introduced in this paper, we have performed a computational experiment. Our goal was to find an example of trees in a database of phylogenetic trees obtained from biological data where our algorithms can be applied.

The general strategy for this search was as follows: We first chose a database with many phylogenetic trees; among these trees we exhaustively searched for a “central” tree sharing many leaves with a large set of “companion” trees in the database.

Then, we exhaustively looked for pairs formed by a subtree of this central tree and a companion tree such that their topological restrictions to their common set of leaves satisfy the principal-secondary condition on clusters.

With all pairs satisfying this condition we looked for a maximal example: with as many leaves as possible and as many secondary trees as possible.

Finally, this maximal set of trees is used as an input to Algorithm 2.

We have taken as our datasource the database of phylogenetic trees in [23]. That database contains 159,905 phylogenetic trees, but in order to make the computations feasible we have restricted our experiment to a random sample of 15,000 trees. Within this sample, we have found a “central” tree *T* with 100 leaves and 200 other “companion” trees sharing at least 30 labels with *T*. We have then kept these 201 trees and discarded the others. Following the strategy described above, we have found the subtree $T_0$ of *T* described by the Newick string

$$\begin{aligned} (((((9,8),7),6),5),((4,3),(1,2))); \end{aligned}$$

where the numbers correspond to the organisms given in

**Table 1:** The organisms involved in the phylogenetic trees $T_0, T_1{^{\prime }},T_2{^{\prime }},T_3{^{\prime }}$ given in §5

Identifier	Organism
1	Roseobacter_denitrificans_OCh_114
2	Ruegeria_pomeroyi_DSS-3
3	Ruegeria_sp._TM1040
4	Dinoroseobacter_shibae_DFL_12
5	Paracoccus_denitrificans_PD1222
6	Rhodobacter_sphaeroides_ATCC_17025
7	Rhodobacter_sphaeroides_KD131
8	Rhodobacter_sphaeroides_ATCC_17029
9	Rhodobacter_sphaeroides_2.4.1

**Fig. 15:**
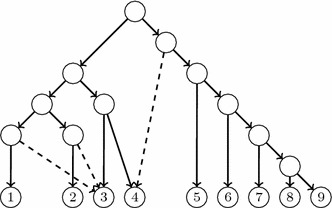
Restricted LGT network obtained in the experiment in §

Table 1, and the following three subtrees of some of the remaining set of 200 trees:

$$\begin{aligned}&T_1{^{\prime }}: (((((9,8),7),6),5),(((2,3),1),4));\\&T_2{^{\prime }}: (((((9,8),7),6),5),(((1,3),2),4));\\&T_3{^{\prime }}: ((((((9,8),7),6),5),4),((3,(1,2)))); \end{aligned}$$

such that each pair of trees $(T_0,T_i{^{\prime }})$, $i=1,2,3$, satisfies the conditions in Proposition 6. Applying Algorithm 2 to $T_0, T_1{^{\prime }},T_2{^{\prime }},T_3{^{\prime }}$, we obtain the restricted LGT network depicted in Fig. 15, that contains $T_0$ as reduced principal subtree and $T_1{^{\prime }},T_2{^{\prime }},T_3{^{\prime }}$ as reduced secondary subtrees. This network suggests the existence of three lateral gene transfer events that explain the differences between $T_0$ and $T_1{^{\prime }},T_2{^{\prime }},T_3{^{\prime }}$. Although there is no reference in the literature to these specific events, several lateral gene transfer events involving *Rhodobacter sp.*, *Ruegeria pom.* and *Ruegeria sp.* have been reported in the literature [33–35].

## Conclusions

In this paper we have considered LGT networks: a general model of phylogenetic networks with lateral gene transfers that capture the asymmetry of these evolutionary events. An LGT network allows to distinguish between the principal line of evolution of the species under study and the secondary lines determined by the lateral gene transfers, by defining, in a natural way, a principal phylogenetic subtree and a family of secondary phylogenetic subtrees.

We have defined a subclass of “restricted” LGT networks such that (a) the principal and secondary phylogenetic subtrees of a restricted LGT network are pairwise different; and (b) the principal and secondary phylogenetic subtrees of a restricted LGT network single it out, up to isomorphisms. Then, we have given an algorithm that solves the problem of reconstructing a restricted LGT network from a given principal phylogenetic subtree and a given family of secondary phylogenetic subtrees, when it exists.

We have implemented the algorithms in this paper using Python. The program can be downloaded from the url http://bioinfo.uib.es/~recerca/LGTnetworks/reconstruction.zip, and the only requirements are the libraries networkx and pyparsing, which are included in most of the standard distributions of python for scientific computation (e.g. anaconda). The zip file contains a README file with specific instructions on how to use the program.

As a future work, we plan to relax the conditions on the restricted LGT networks in order to be able to reconstruct a broader class of networks and discover new algorithms for reconstructing such networks from biologically significant data.

## Availability

The Python program implementing our algorithms is available at http://bioinfo.uib.es/~recerca/LGTnetworks/reconstruction.zip

## Additional file

10.1007/s13015-015-0059-z Appendix: Some proofs

## Footnotes

1 We omit the ending semicolon in order not to unnecessarily overload the triples’ notation.

2 Henceforth, in graphical representations of LGT networks, we shall use the following conventions: principal arcs are represented by continuous arrows, secondary arcs by dashed arrows, and principal paths by continuous snaked arrows.
