# Supplementary material for: A reconstruction problem for a class of phylogenetic networks with lateral gene transfers
Source: Algorithms Mol Biol. 2015 Dec 2;10:28. doi: 10.1186/s13015-015-0059-z (PMC4683721; doi:10.1186/s13015-015-0059-z)
Supplement: Supplementary file 1 — 10.1007/s13015-015-0059-z Appendix: Some proofs [file 13015_2015_59_MOESM1_ESM.pdf]

RESEARCH

A reconstruction problem for a class of phylogenetic networks with lateral gene transfers

Gabriel Cardona[*], Joan Carles Pons[†] and Francesc Rosselló

[*]Correspondence:
gabriel.cardona@uib.es
Dept. of Mathematics and Computer Science, University of the Balearic Islands, E-07122 Palma de Mallorca, Spain
Full list of author information is available at the end of the article
[†]Corresponding author

Appendix: Some proofs

Proof of Proposition 4

Let $e = (u, h), e' = (u', h') \in E_s$; to simplify the notations, we shall denote $T_0(N)$, $T_e(N)$ and $T_{e'}(N)$ by T_0, T_e and $T_{e'}$, respectively. We shall prove that $\Gamma(T_e) \neq \Gamma(T_{e'})$, which will imply, by Proposition 2, that $\widetilde{T}_e \neq \widetilde{T}_{e'}$. To do that, we shall distinguish three main cases, depending on the relationship between u and u' in T_0, and in each case (and its subcases, when necessary) we shall show the existence of three labels on which T_e and $T_{e'}$ define different triples.

A) Consider first the case when $u = u'$. By condition (c) in Definition 2, neither h nor h' are principal descendants of u, and therefore $C_{T_0}(u) \cap C_{T_0}(h) = C_{T_0}(u) \cap C_{T_0}(h') = \emptyset$. This implies the existence of a leaf $x_3 \in C_{T_0}(u)$ that does not belong to $C_{T_0}(h) \cup C_{T_0}(h')$.

Since there cannot exist simultaneously two principal paths $h \rightsquigarrow h'$ and $h' \rightsquigarrow h$, we shall assume without any loss of generality that the latter, $h' \rightsquigarrow h$, does not exist. If there exists a principal path $h \rightsquigarrow h'$, then, since h is not principally elementary by condition (b) in Definition 2, it has a principal child v outside this principal path and then any leaf $x_1 \in C_{T_0}(v)$ belongs to $C_{T_0}(h)$ but not to $C_{T_0}(h')$. If, on the contrary, no principal path connects h with h', then $C_{T_0}(h) \cap C_{T_0}(h') = \emptyset$ and no $x_1 \in C_{T_0}(h)$ belongs to $C_{T_0}(h')$. So, in both cases, there exists some leaf $x_1 \in C_{T_0}(h) \setminus C_{T_0}(h')$. Take, finally, $x_2 \in C_{T_0}(h')$; see Fig. 16. Notice that $x_1, x_2 \notin C_{T_0}(u)$.

Now, on the one hand, in $T_{e'}$ we have that $u = LCA_{T_{e'}}(x_2, x_3)$ and $x_1 \notin C_{T_{e'}}(u) = C_{T_0}(h') \cup C_{T_0}(u)$. This implies that $T_{e'}$ defines the triple $((x_2, x_3), x_1)$. On the other hand, in T_e, the principal path $u \rightsquigarrow x_3$ survives because h is not a principal descendant of u, and therefore $u = LCA_{T_{e'}}(x_1, x_3)$, and moreover, since h' is nor a principal descendant of u, there cannot be any path in T_e from an intermediate node in the principal path $u \rightsquigarrow x_3$ to x_2. This makes it impossible that T_e defines the triple $((x_2, x_3), x_1)$.

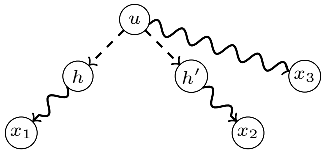

Figure 16 The structure of N involving e and e' in case (A) in the proof of Prop. 4.

B) Consider now the case when u and u' are connected by a proper principal path, say $u \rightsquigarrow u'$. By condition (c) in Definition 2, there do not exist principal paths connecting u with h or u' with either h' or h.

Assume first that the principal path $u \rightsquigarrow u'$ is not principally elementary. Let v be the last node in this path that is not principally elementary (v can be u, if every intermediate node in the path $u \rightsquigarrow u'$ is principally elementary), and let v' be any principal child of v outside this path $u \rightsquigarrow u'$. If h' is not a principal descendant of v', take $x_3 \in C_{T_0}(v')$. If, on the contrary, h' is a principal descendant of v', then, by condition (d) in Definition 2, the principal path $v \to v' \rightsquigarrow h'$ must contain some intermediate node w with a principal child w' outside this path; in this case, take $x_3 \in C_{T_0}(w')$. In this way, we always obtain a leaf $x_3 \in C_{T_0}(v) \setminus (C_{T_0}(u') \cup C_{T_0}(h'))$. Let, moreover, $x_1 \in C_{T_0}(u')$ and $x_2 \in C_{T_0}(h')$; notice that $x_1 \neq x_2$ because $C_{T_0}(u') \cap C_{T_0}(h') = \emptyset$. The situation is summarized in Fig. 17.(a).

Since h' does not belong to the principal paths $v \rightsquigarrow x_3$ or $v \rightsquigarrow u' \rightsquigarrow x_1$, it is clear that $T_{e'}$ defines the triple $((x_1, x_2), x_3)$. Let us prove now that T_e cannot define this triple. Indeed, notice that, since h is not a principal descendant of u, it does not belong to the principal paths $v \rightsquigarrow x_3$ or $v \rightsquigarrow u' \rightsquigarrow x_1$. This implies that these paths survive in T_e and hence that $LCA_{T_e}(x_1, x_3) = v$. Therefore, should T_e define the triple $((x_1, x_2), x_3)$, this would imply that T_e contains some path $u' \rightsquigarrow x_2$ (recall that every intermediate node in the principal path $v \rightsquigarrow u'$ is principally elementary). But since u cannot be a descendant of u', this path could not contain the secondary arc $e = (u, h)$ and therefore it would be principal, implying the existence of a principal path connecting u' and h', which does not exist. This leads to a contradiction, showing that, as we claimed, T_e does not define the triple $((x_1, x_2), x_3)$.

Assume now that the principal path $u \rightsquigarrow u'$ is principally elementary. Since T_0 cannot contain two consecutive elementary nodes, this implies that u' is not principally elementary (and that $(u, u') \in E_p$). Let u'_1, u'_2 be two principal children of it and let $x_1 \in C_{T_0}(u'_1)$ and $x_2 \in C_{T_0}(u'_2)$, and let $x_3 \in C_{T_0}(h')$; see Fig. 17.(b). Then $T_{e'}$ defines the triple (x_1, x_2, x_3). Now, since h is not a principal descendant of u, the bifurcating principal paths $u' \rightsquigarrow x_1$ and $u' \rightsquigarrow x_2$ survive in T_e and hence $LCA_{T_e}(x_1, x_2) = u'$, but h' is not a descendant of u' in T_e (because neither h' nor u are principal descendants of u') and therefore T_e defines the triple $((x_1, x_2), x_3)$.

In both cases, $\Gamma(T_e) \neq \Gamma(T_{e'})$.

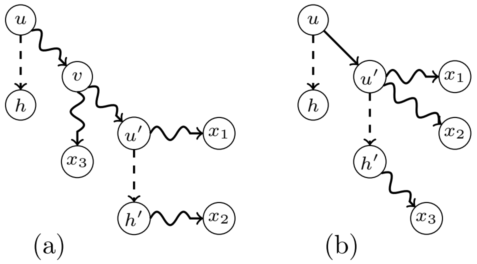

Figure 17 The two possible structures of N involving e and e' in case (B) in the proof of Prop. 4.

C) Assume finally that u and u' are different and not connected by any principal path, that is, $C_{T_0}(u) \cap C_{T_0}(u') = \emptyset$. Recall that, by condition (c) in Definition

2, $C_{T_0}(u) \cap C_{T_0}(h) = C_{T_0}(u') \cap C_{T_0}(h') = \emptyset$, too. We shall consider now several subcases, up to interchanging e and e'.

C.1) Assume that there exist principal paths $u \rightsquigarrow h'$ and $h \rightsquigarrow u'$. Then, since $C_{T_0}(h') \subseteq C_{T_0}(u)$ and $C_{T_0}(u) \cap C_{T_0}(h) = \emptyset$, we have that $C_{T_0}(h) \cap C_{T_0}(h') = \emptyset$. Moreover, since h is not principally elementary, there exists some leaf $x_1 \in C_{T_0}(h) \setminus C_{T_0}(u')$. Take any $x_2 \in C_{T_0}(h')$ and $x_3 \in C_{T_0}(u')$ (see Fig. 18, where $v = LCA_{T_0}(u,h)$). It is easy to check that T_e defines the triple $((x_1,x_3),x_2)$ and $T_{e'}$ defines the triple $((x_2,x_3),x_1)$.

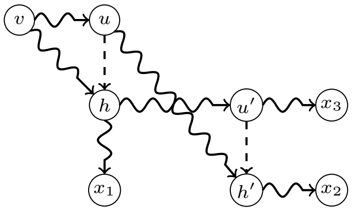

Figure 18 The structure of N involving e and e' in case (C.1) in the proof of Prop. 4.

C.2) Assume that there exist principal paths $u \rightsquigarrow h'$ and $u' \rightsquigarrow h$. As in (C.1), this implies that $C_{T_0}(h) \cap C_{T_0}(h') = \emptyset$. Let $v = LCA_{T_0}(u,u')$. By condition (d) in Definition 2, the principal path $v \rightsquigarrow u \rightsquigarrow h'$ contains some intermediate node w with a principal child w_1 outside this path, and the principal path $v \rightsquigarrow u' \rightsquigarrow h$ contains some intermediate node w' with a principal child w'_1 outside this path.

Assume first that, up to interchanging u and u', the node w belongs the path $u \rightsquigarrow h'$ (this includes the case $w = u$). Let $x_1 \in C_{T_0}(w_1)$, $x_2 \in C_{T_0}(h)$ and $x_3 \in C_{T_0}(h')$; see Fig. 19.(a) (where w can be u; we have not distinguished this possibility in the figure). In this case, T_e defines the triple $((x_1,x_3),x_2)$ and $T_{e'}$ defines the triple $((x_2,x_3),x_1)$.

Assume now that both principal paths $u \rightsquigarrow h'$ and $u' \rightsquigarrow h$ are principally elementary; that is, principal arcs with u and u' principally elementary. In this case, w is intermediate in the principal path $v \rightsquigarrow u$ and w' is intermediate in the principal path $v \rightsquigarrow u'$. Let $x_1 \in C_{T_0}(w_1)$, $x_2 \in C_{T_0}(w'_1)$ and $x_3 \in C_{T_0}(h)$; see Fig. 19.(b). Then, T_e defines the triple $((x_1,x_3),x_2)$ and $T_{e'}$ defines the triple $((x_2,x_3),x_1)$.

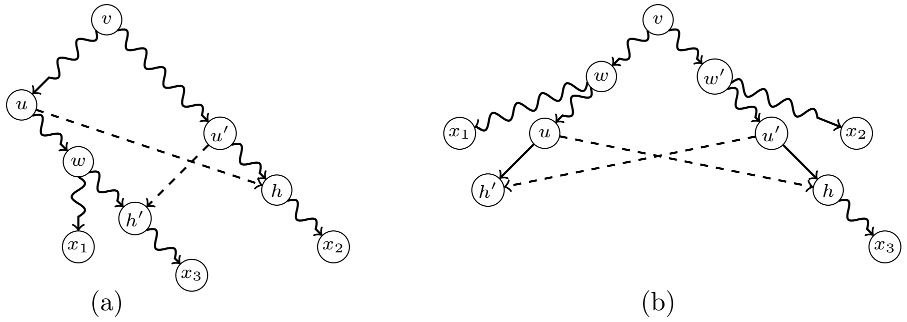

Figure 19 The structure of N involving e and e' in case (C.2) in the proof of Prop. 4.

C.3) Assume that there exist a principal path $u \rightsquigarrow h'$ but no principal path connecting u' and h. Since u and h are not connected by any principal path, neither

are h and h'. Let $v = LCA_{T_0}(u,u')$, $x_1 \in C_{T_0}(h)$, $x_2 \in C_{T_0}(h')$ and $x_3 \in C_{T_0}(u')$ (see Fig. 20). In $T_{e'}$, we have that $u' = LCA_{T_{e'}}(x_2,x_3)$ and, since there exists no principal path connecting h with u' or h', $x_1 \notin C_{T_{e'}}(u')$. This implies that $T_{e'}$ defines the triple $((x_2,x_3),x_1)$. Now, again because h is not connected in T_0 with u' or h', the principal paths $u \rightsquigarrow h' \rightsquigarrow x_2$ and $v \rightsquigarrow u' \rightsquigarrow x_3$ survive in T_e, and hence this tree defines the triple $((x_1,x_2),x_3)$.

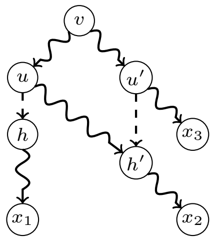

Figure 20 The structure of N involving e and e' in case (C.3) in the proof of Prop. 4.

C.4) Assume that there exists no principal path from $\{u,u'\}$ to $\{h,h'\}$, but there exists a principal path $h \rightsquigarrow u'$. Let $v = LCA_{T_0}(u,h) = LCA_{T_0}(u,u')$.

By condition (d) in Definition 2, the principal path $v \rightsquigarrow h$ contains some intermediate node w with a principal child w_1 outside this path. If h' is not a principal descendant of w_1, take $x_1 \in C_{T_0}(w_1)$. If h' is a principal descendant of w_1, then $w = LCA_{T_0}(u',h')$, and the path $w \to w_1 \rightsquigarrow h'$ contains some intermediate node w' with a principal child w'_1 outside this path: in this case, take $x_1 \in C_{T_0}(w'_1)$. In both cases, $x_1 \in C_{T_0}(w)$ and $x_1 \notin C_{T_0}(h) \cup C_{T_0}(h')$. Let moreover $x_2 \in C_{T_0}(u)$ and $x_3 \in C_{T_0}(u')$: see Fig. 21.

It is clear then that T_e defines the triple $((x_2,x_3),x_1)$. Now, h' does not belong to the principal paths $v \rightsquigarrow h \rightsquigarrow u' \rightsquigarrow x_3$, $w \rightsquigarrow x_1$ or $v \rightsquigarrow u \rightsquigarrow x_2$ (as far as this last path goes, notice that u cannot be a principal descendant of h', because h' is a descendant of u in N, and that h' is not a principal descendant of u by assumption). Therefore, these principal paths survive in $T_{e'}$ and this tree defines the triple $((x_1,x_3),x_2)$.

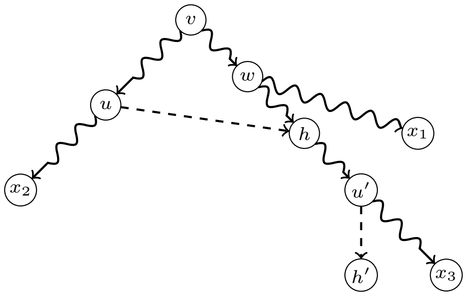

Figure 21 The structure of N involving e and e' in case (C.4) in the proof of Prop. 4.

C.5) Assume now that there exists no principal path connecting a node in $\{u,u'\}$ and a node in $\{h,h'\}$, but that h and h' are connected by a principal path, say $h \rightsquigarrow h'$ (this includes the case $h = h'$). Let $v = LCA_{T_0}(u,u')$, $x_1 \in C_{T_0}(u)$, $x_2 \in C_{T_0}(u')$, and $x_3 \in C_{T_0}(h')$; see Fig. 22. Then, T_e defines the triple $((x_1,x_3),x_2)$ and $T_{e'}$ defines the triple $((x_2,x_3),x_1)$.

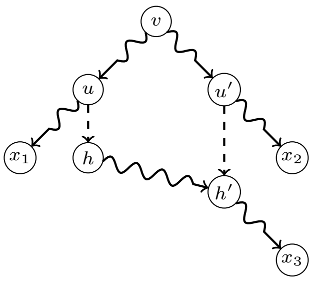

Figure 22 The structure of N involving e and e' in case (C.5) in the proof of Prop. 4. The path $h \rightsquigarrow h'$ need not be proper.

C.6) Assume finally that there exists no principal path connecting any pair of nodes $\{u, u', h, h'\}$. Let $v = LCA_{T_0}(u, u')$. We shall split this case into two subcases, up to symmetry.

Assume first that $w = LCA_{T_0}(u, h)$ is not a proper descendant of v: therefore, it is an ancestor of it. In this case, let $x_1 \in C_{T_0}(u)$, $x_2 \in C_{T_0}(h)$, and $x_3 \in C_{T_0}(u')$; see Fig. 23.(a) (where w can be v; we have not distinguished this possibility in the figure). Since h does not belong to the principal path $v \rightsquigarrow u' \rightsquigarrow x_3$, T_e defines the triple $((x_1, x_2), x_3)$, and since h' does not belong to the principal paths $w \rightsquigarrow v \rightsquigarrow u \rightsquigarrow x_1$, $w \rightsquigarrow h \rightsquigarrow x_2$ and $w \rightsquigarrow v \rightsquigarrow u' \rightsquigarrow x_3$, they survive in $T_{e'}$ and then it defines either the triple $((x_1, x_3), x_2)$ (if $v \neq w$) or (x_1, x_2, x_3) (if $v = w$).

Assume now that $w = LCA_{T_0}(u, h)$ and $w' = LCA_{T_0}(u', h')$ are both proper descendants of v and therefore they are intermediate nodes in the principal paths $v \rightsquigarrow u$ and $v \rightsquigarrow u'$, respectively; in particular, $v = LCA_{T_0}(h, h')$. By condition (d) in Definition 2, the path $w \rightsquigarrow h$ must contain some intermediate node w_0 with some principal child w_1 outside this path. Let $x_3 \in C_{T_0}(w_1)$, $x_1 \in C_{T_0}(u)$, and $x_2 \in C_{T_0}(h)$: see Fig. 23.(b).

In this situation, T_e defines the triple $((x_1, x_2), x_3)$ and (since h' cannot belong to the principal path $w_1 \rightsquigarrow x_3$, because $LCA_{T_0}(h, h') = v$) $T_{e'}$ defines the same triple on x_1, x_2, x_3 as T_0, namely $((x_2, x_3), x_1)$.

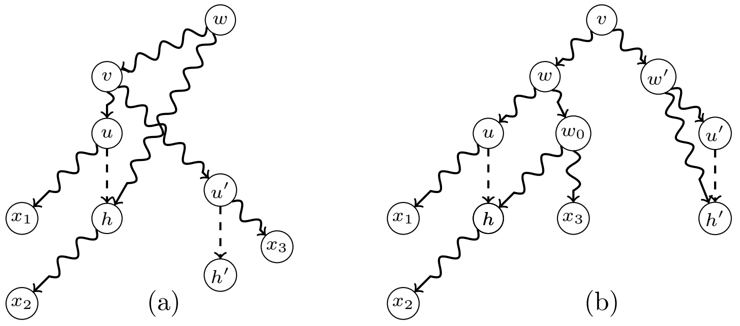

Figure 23 The structure of N involving e and e' in case (C.6) in the proof of Prop. 4.

So, $\Gamma(T_e) \neq \Gamma(T_{e'})$ in all subcases in which we have divided (C). This finishes the proof that T_e and $T_{e'}$ always define different sets of triples. □

Proof of the Claims in Proposition 6

We first establish an easy auxiliary lemma on triples calculus, which will allow us to avoid repeating the same argument several times:

Lemma 1 *Let T a phylogenetic tree on S, and let $x, y, z, t \in S$.*
(1) If $((x, y), z), ((x, t), z) \in \Gamma(T)$, then $((y, t), z) \in \Gamma(T)$.
(2) If $((x, y), z), ((z, t), x) \in \Gamma(T)$, then $((x, y), t) \in \Gamma(T)$.
(3) If $((x, y), z) \in \Gamma(T)$ and $((x, y), t) \notin \Gamma(T)$, then $((x, t), z) \in \Gamma(T)$.

Proof Assertions (1) and (2) are proved in [1, Cor. 9.3]; although the trees considered therein are binary, it is easy to check that the proof is valid in the arbitrary setting. As far as (3) goes, if $((x, y), z) \in \Gamma(T)$, then $LCA_T(x, y)$ is a proper descendant of $LCA_T(x, z)$. Now, if moreover $((x, y), t) \notin \Gamma(T)$, then t is a descendant of $LCA_T(x, y)$ and hence $LCA_T(x, t)$ is a descendant of $LCA_T(x, y)$ and *a fortiori* a proper descendant of $LCA_T(x, z)$, which implies that $((x, t), z) \in \Gamma(T)$. □

Let us proceed with the proofs of the claims. Assume in the rest of this appendix that $\Gamma(T) \triangle \Gamma(T')$ consists of those triples described in the statement. To simplify the notations, set $A = \bigcup_{i=1}^{k} A_i$, $C_l = \bigcup_{i=1}^{m} C_{l,i}$ and $C = \bigcup_{i=1}^{l} C_i$.

Claim 1: $B \in C(T) \cap C(T')$.

We shall prove that, for every $b, b' \in B$ and $x \notin B$, $((b, b'), x) \in \Gamma(T) \cap \Gamma(T')$, which implies that $B \in C(T) \cap C(T')$. Since, by the explicit description of $\Gamma(T) \triangle \Gamma(T')$ given in the statement, $T_{b,b',x} = T'_{b,b',x}$, it is enough to prove that $((b, b'), x) \in \Gamma(T) \cup \Gamma(T')$. To do that, we consider three different cases, depending on x.

(1.1) If $x \in A$, let $c \in C$. Since $((b, c), x), ((b', c), x) \in \Gamma(T')$, by Lemma 1.(1) we have that $((b, b'), x) \in \Gamma(T')$.

(1.2) If $x \in C$, let $a \in A$. Since $((a, b), x), ((a, b'), x) \in \Gamma(T)$, again by Lemma 1.(1) we have that $((b, b'), x) \in \Gamma(T)$.

(1.3) If $x \notin A \cup B \cup C$, let $a \in A$ and $c \in C$. We shall assume that $((b, b'), x) \notin \Gamma(T) \cup \Gamma(T')$ and we shall reach a contradiction. Indeed, we know from (1.1) that $((b, b'), a) \in \Gamma(T')$. Then, if $((b, b'), x) \notin \Gamma(T')$, by Lemma 1.(3), we have that $((b, x), a) \in \Gamma(T')$, and since $((b, c), a) \in \Gamma(T')$, Lemma 1.(1) implies that $((x, c), a) \in \Gamma(T')$. On the other hand, we know from (1.2) that $((b, b'), c) \in \Gamma(T)$. Then, if $((b, b'), x) \notin \Gamma(T)$, by Lemma 1.(3) we have that $((b, x), c) \in \Gamma(T)$, and since $((a, b), c) \in \Gamma(T)$, by Lemma 1.(1) we have that $((a, x), c) \in \Gamma(T)$. But if $x \notin B$, $T_{a,c,x} = T'_{a,c,x}$, which leads to the announced contradiction.

This finishes the proof of Claim 1.

Claim 2. $C_{l,i} \in C(T) \cap C(T')$, for every $i = 1, \ldots, m$.

We shall prove that, for every $c, c' \in C_{l,i}$ and $x \notin C_{l,i}$, $((c, c'), x) \in \Gamma(T) \cap \Gamma(T')$. As in the previous Claim, it is enough to prove that $((c, c'), x) \in \Gamma(T) \cup \Gamma(T')$; and, also as in the previous claim, we consider different possibilities for x.

(2.1) If $x \in A \cup \bigcup_{i<l} C_i$, let $b \in B$. Since $((b, c), x), ((b, c'), x) \in \Gamma(T')$, by Lemma 1.(1) we have that $((c, c'), x) \in \Gamma(T')$.

(2.2) If $x \in B$, let $a \in A$. As we have just seen, $((c,c'),a) \in \Gamma(T')$ and therefore $((c,c'),a) \in \Gamma(T)$. Then, since $((a,x),c) \in \Gamma(T)$, by Lemma 1.(2) we have that $((c,c'),x) \in \Gamma(T)$.

(2.3) If $x \in C_{l,j}$ for some $j \neq i$, let $b \in B$. By (2.2), $((c,c'),b) \in \Gamma(T)$ and hence $((c,c'),b) \in \Gamma(T')$, too; moreover, by assumption, $(c,x,b),(c',x,b) \in \Gamma(T')$. Then, $LCA_{T'}(x,c) = LCA_{T'}(b,c) = LCA_{T'}(b,x) = LCA_{T'}(b,c') = LCA_{T'}(x,c')$ and it is a proper ancestor of $LCA_{T'}(c,c')$, which implies that $((c,c'),x) \in \Gamma(T')$.

(2.4) If $x \notin A \cup B \cup C$, let $a \in A$ and $b \in B$. We shall assume that $((c,c'),x) \notin \Gamma(T) \cup \Gamma(T')$ and we shall reach a contradiction. Indeed, by (2.1) we know that $((c,c'),a) \in \Gamma(T')$, and therefore, if $((c,c'),x) \notin \Gamma(T')$, Lemma 1.(3) implies that $((c,x),a) \in \Gamma(T')$. Moreover, $((b,c),a) \in \Gamma(T')$ and then, by Lemma 1.(1), $((b,x),a) \in \Gamma(T')$. Since T and T' define the same triples on $\{a,c,c'\},\{a,c,x\},\{a,b,x\}$, we have that $((c,c'),a),((c,x),a),((b,x),a) \in \Gamma(T)$. But $\Gamma(T)$ also contains $((a,b),c)$, and this is impossible, because these four triples are incompatible: the Aho graph they define on $\{a,b,c,c',x\}$ is connected [2].

This finishes the proof of Claim 2.

Claim 3. $B \cup C_l \in C(T') \setminus C(T)$.

Since, for every $a \in A$, $b \in B$ and $c \in C_l$, $T_{a,b,c} = ((a,b),c)$, it is clear that $B \cup C_l$ is not a cluster of T. Now, to prove that $B \cup C_l \in C(T')$, since we already know that $B \in C(T')$, we shall prove that:

(i) For every $c,c' \in C_l$ and $x \notin B \cup C_l$, T' defines the triple $((c,c'),x)$.

(ii) For every $b \in B$, $c \in C_l$ and $x \notin B \cup C_l$, T' defines the triple $((b,c),x)$.

As far as (i) goes, we distinguish the following cases:

(3.1) If $x \in A \cup \bigcup_{i<l} C_i$, the same argument as in (2.1) proves that $((c,c'),x) \in \Gamma(T')$.

(3.2) If $x \notin A \cup B \cup C$, the same argument as in (2.4) proves that $((c,c'),x) \in \Gamma(T')$.

As far as (ii) goes, we distinguish the following cases:

(3.3) If $x \in A \cup \bigcup_{i<l} C_i$, we already know by assumption that $((b,c),x) \in \Gamma(T')$.

(3.4) If $x \notin A \cup B \cup C$, let $a \in A$; we shall assume that $((b,c),x) \notin \Gamma(T')$ and we shall reach a contradiction. Indeed, if this is the case, since $((b,c),a) \in \Gamma(T')$, by Lemma 1.(3) we obtain that $((b,x),a),((c,x),a) \in \Gamma(T')$. Since $T_{a,b,x} = T'_{a,b,x}$ and $T_{a,c,x} = T'_{a,c,x}$, this implies that $((b,x),a),((c,x),a) \in \Gamma(T)$, but then, by Lemma 1.(1), this implies that $((b,c),a) \in \Gamma(T)$, which contradicts the assumption that $((a,b),c) \in \Gamma(T)$.

This finishes the proof of Claim 3.

Claim 4. If $m > 1$, $C_l \in C(T) \setminus C(T')$.

C_l is not a cluster in T' when $m > 1$, because if $c \in C_{l,1}$, $c' \in C_{l,2}$ and $b \in B$, then $T'_{c,c',b} = (c,c',b)$ and therefore $((c,c'),b) \notin \Gamma(T')$. To prove that $C_l \in C(T)$, it is enough to check that, for every $c,c' \in C_l$ and $x \notin C_l$, $((c,c'),x) \in \Gamma(T)$. Now:

(4.1) If $x \in A \cup \bigcup_{i<l} C_i$, by (3.1) we know that $((c,c'),x) \in \Gamma(T')$, and, by assumption, in this case $T'_{c,c',x} = T_{c,c',x}$.

(4.2) If $x \in B$, the same argument as in (2.2) proves that $((c,c'),x) \in \Gamma(T)$.

(4.3) If $x \notin A \cup B \cup C$, by (3.2) we know that $((c,c'),x) \in \Gamma(T')$, and, by assumption, in this case $T'_{c,c',x} = T_{c,c',x}$, too.

This finishes the proof of Claim 4.

Claim 5. If $m > 1$, $B \cup C_{l,i_1} \cup \cdots \cup C_{l,i_k} \notin C(T')$ for every $\emptyset \neq \{i_1, \ldots, i_k\} \subsetneq \{1, \ldots, m\}$

Let $c \in \bigcup_{j=1}^{k} C_{l,i_j}$, $c' \in C_l \setminus \bigcup_{j=1}^{k} C_{l,i_j}$ and $b \in B$. Then, by assumption, $T'_{c,c',b} = (c, c', b)$ and therefore $((c, b), c') \notin \Gamma(T')$.

This finishes the proof of Proposition 6. □

Proof of Proposition 8

As far as the "only if" implication goes, assume that $e = (w, h)$ and let $T = \widetilde{T}_0(N)$ and $T' = \widetilde{T}_e(N)$. Consider the situation of the corresponding implication in Proposition 6, depicted in Fig. 9: we shall use the same notations as therein.

- For every $i = 1, \ldots, k$, let $U_i = C_T(u_i)$.
- For every $i = 1, \ldots, k-1$, let $U'_i = C_{T'}(u_i)$; if, moreover, $\mathrm{outdeg}_T(u_k) > 2$, let $U'_k = A_k$, which then belongs to $C(T') \setminus C(T)$.
- For every $i = 1, \ldots, l$, let $W'_i = C_{T'}(w_i)$ (and recall that $w_l = \tilde{w}$, the first non principally elementary principal descendant of w).
- For every $i = 1, \ldots, l-1$, let $W_i = C_T(w_i)$; if, moreover, $\tilde{w} = w$ (that is, if $\mathrm{outdeg}_{T_0(N)}(w) \geqslant 2$), let $W_l = C_l$, which then belongs to $C(T) \setminus C(T')$.

It is straightforward to check that conditions (a) to (f) in the statement are satisfied.

In order to prove the "if" implication, assume that $C(T)$ and $C(T')$ satisfy conditions (a) to (f) in the statement. Let B, A_k, C_l be as defined in conditions (b)–(d), let $U'_k = A_k$ and $W_l = C_l$ even when $k_0 = k-1$ or $l_0 = l-1$ (the only difference is that, in these cases, they belong to $C(T) \cap C(T')$), and let

$$A_i := U_i \setminus U_{i+1} = U'_i \setminus U'_{i+1}, \text{ for every } i = 1, \ldots, k-1$$
$$C_i := W_i \setminus W_{i+1} = W'_i \setminus W'_{i+1}, \text{ for every } i = 1, \ldots, l-1$$

It is easy to check, then, that

$$U_i = A_i \sqcup \cdots \sqcup A_k \sqcup B \text{ for every } i = 1, \ldots, k$$
$$U'_i = A_i \sqcup \cdots \sqcup A_k \text{ for every } i = 1, \ldots, k$$
$$W_i = C_i \sqcup \cdots \sqcup C_l \text{ for every } i = 1, \ldots, l$$
$$W'_i = C_i \sqcup \cdots \sqcup C_l \sqcup B \text{ for every } i = 1, \ldots, l$$

so that, in particular, $U'_i = U_i \setminus B$, for every $i = 1, \ldots, k$, and $W_i = W'_i \setminus B$, for every $i = 1, \ldots, l$.

Let h' the node in T' with cluster B. If $l_0 = l$ (that is, if $C_l \notin C(T')$), let w' be the parent of h', let $x'_1, \ldots, x'_m$ be the other children of w' and let $C_{l,i} = C_{T'}(x'_i)$, for every $i = 1, \ldots, m$.

It turns out that, with these notations, the symmetric difference $\Gamma(T) \triangle \Gamma(T')$ consists exactly of those triples described in the statement of Proposition 6. To prove it, we shall describe explicitly the structures of T and T':

- Let h be the node in T with $C_T(h) = B$; recall that h' is the node in T' with $C_{T'}(h') = B$.

- For every $i = 1, \ldots, k$, let u_i be the node in T such that $U_i = C_T(u_i)$ and, for every $i = 1, \ldots, k-1$, let u'_i be the node in T' such that $U'_i = C_{T'}(u'_i)$. By (a), u_{i+1} is a child of u_i for every $i = 1, \ldots, k-1$, and u'_{i+1} is a child of u'_i for every $i = 1, \ldots, k-2$.
- Let u'_k be the node in T' such that $C_{T'}(u'_k) = U'_k = A_k$, which exists by (c), and assume that $k > 1$. Then, since $A_k \subsetneq U'_{k-1}$ by (e), u'_k is a descendant of u'_{k-1}. It turns out that u'_k is a child of u'_{k-1}.
  Indeed, if $k_0 = k$, then it is a direct consequence of (a). Assume now that $k_0 = k-1$, so that the cluster of every proper descendant of u'_{k-1} also belongs to $C(T)$. If u'_k is not a child of u'_{k-1}, the path $u'_{k-1} \rightsquigarrow u'_k$ contains an intermediate node $\bar{u}$ with $C_{T'}(\bar{u}) = A_k \sqcup \bar{A}$, with $\emptyset \neq \bar{A} \subsetneq A_{k-1}$. Then, $A_k \sqcup \bar{A} \in C(T)$ and $U_k \cap (A_k \sqcup \bar{A}) = (A_k \sqcup B) \cap (A_k \sqcup \bar{A}) \neq \emptyset$. This implies, by the compatibility of clusters in phylogenetic trees, that either $B \subseteq \bar{A}$ or $\bar{A} \subseteq B$ and hence that $\emptyset \neq B \cap A_{k-1}$, which is false. This contradiction implies that $\bar{u}$ cannot exist, and therefore that u'_k is a child of u'_{k-1}, as we claimed.
- For every $i = 1, \ldots, l-1$, let w_i be the node in T such that $W_i = C_T(w_i)$ and, for every $i = 1, \ldots, l$, let w'_i be the node in T' such that $W'_i = C_{T'}(w'_i)$. Again by (a), w_{i+1} is a child of w_i for every $i = 1, \ldots, l-2$, and w'_{i+1} is a child of w'_i for every $i = 1, \ldots, l-1$.
- Let w_l be the node in T such that $C_T(w_l) = W_l = C_l$, which exists by (d). A similar argument as the one used to prove that u'_k is a child of u'_{k-1} also proves that, if $l > 1$, w_l is a child of w_{l-1}.
- Let us prove now that h is a child of u_k. If $k_0 = k-1$, it is a direct consequence of the fact that $A_k \in C(T)$ by (c): if, in this case, a_k is the node in T such that $C_T(a_k) = A_k$, then the equality $U_k = A_k \sqcup B$ implies that a_k and h are the only children of u_k.
  Assume now that $k_0 = k$. Since $B \subsetneq U_k$, h is a proper descendant of u_k. Assume that the path $u_k \rightsquigarrow h$ contains some intermediate node $\bar{u}$, with $C_T(\bar{u}) = B \sqcup \bar{A}$ with $\emptyset \neq \bar{A} \subsetneq A_k$. By (a), the cluster of every proper descendant of u_k also belongs to $C(T')$, and therefore $B \sqcup \bar{A} \in C(T')$. But then, $W'_l = B \sqcup C_l \in C(T')$ by (d) and $(B \sqcup \bar{A}) \cap (B \sqcup C_l) \neq \emptyset$, which implies that $\bar{A} \subseteq C_l$ or $C_l \subseteq \bar{A}$ and hence $C_l \cap \bar{A} \neq \emptyset$, which is impossible because $C_l \cap \bar{A} \subseteq C_l \cap A_k \subseteq W'_1 \cap U'_1 = \emptyset$. This contradiction implies that $\bar{u}$ cannot exist, and hence that h is a child of u_k, as we claimed.
- A similar argument shows that h' is a child of w'_l. In particular, $w' = w'_l$.
- The equalities $U_1 \cap W_1 = W'_1 \cap U'_1 = \emptyset$ always hold. Indeed
  - if $k, l > 1$ or $k_0 = k = 1$ or $l_0 = l = 1$, these intersections are empty by (a);
  - if $k > 1$, $l = 1$ and $l_0 = l - 1$, then $W'_1 \cap U'_1 = \emptyset$ by (a) and $U_1 \cap W_1 = (U'_1 \sqcup B) \cap C_1 = (U'_1 \sqcup B) \cap (W'_1 \setminus B) = \emptyset$;
  - if $l > 1$, $k = 1$ and $k_0 = k - 1$, a symmetrical argument applies;
  - if $k = l = 1$, $k_0 = k-1$, then $C(T) \setminus C(T') = \{U_1\}$ with $U_1 = A_1 \sqcup B$ and $C(T') \setminus C(T) = \{W'_1\}$ with $W'_1 = C_1 \sqcup B$. Now, in this case, $U_1 \cap W_1 = W'_1 \cap U'_1 = A_1 \cap C_1 = \emptyset$, because, by (b), $(A_1 \sqcup B) \cap (C_1 \sqcup B) = U_1 \cap W'_1 = B$.
- $U_1 \cup W_1 = W'_1 \cup U'_1$: actually, in all cases,

$$U_1 \cup W_1 = A_1 \sqcup \cdots \sqcup A_k \sqcup B \sqcup C_1 \sqcup \cdots \sqcup C_l = W'_1 \sqcup U'_1.$$

- Let us check that, if $l > 1$ or $l_0 = l = 1$, the nodes u_1 and w_1 are sibling in T. To do that, recall that, by (a), every cluster in T strictly containing U_1 or W_1 is also a cluster in T'.
  Let v be any proper ancestor of u_1. Then, $C_T(v) = U_1 \sqcup X$ with $X \neq \emptyset$, and, thus, $U_1 \sqcup X \in C(T')$. Now, since $(U_1 \sqcup X) \cap U_1' \neq \emptyset$ and $(U_1 \sqcup X) \cap W_1' \neq \emptyset$ (because $U_1 = U_1' \sqcup B$ and $B \subsetneq W_1'$) and $U_1' \cap W_1' = \emptyset$, we conclude that $U_1' \cup W_1' \subseteq U_1 \sqcup X$ and in particular $W_1 \subsetneq W_1' \subseteq U_1 \sqcup X$. This entails that every proper ancestor of u_1 is also an ancestor of w_1.
  Let now v be any proper ancestor of w_1. Then, $C_T(v) = W_1 \sqcup X$ with $X \neq \emptyset$ and, hence, $W_1 \sqcup X \in C(T')$. Now, since $(W_1 \sqcup X) \cap W_1' \neq \emptyset$ and $W_1' = W_1 \sqcup B$, we conclude that $X \cap B \neq \emptyset$ and hence $(W_1 \sqcup X) \cap U_1 \neq \emptyset$. Since $W_1 \cap U_1 = \emptyset$, this implies that $U_1 \subseteq X$ and, hence, that u_1 is a descendant of v. Therefore, every proper ancestor of w_1 is also an ancestor of u_1. This finishes the proof that u_1 and v_1 are sibling.
- A similar argument shows that, if $k > 1$ or $k_0 = k = 1$, the nodes u_1' and w_1' are sibling in T'.
- Assume now that $l_0 = l$; as we have seen, the parent w' of h' is w_l'. In this case, by (d), $C_{T'}(w_l') = W_l' = C_l \sqcup B$ and $C_l \notin C(T)$, which means that w_l' has more than one child other than h'. Consider the clusters $C_{l,1}, \ldots, C_{l,m} \in C(T')$, with $m > 1$, of the children of w_l' other than h', so that $C_l = C_{l,1} \sqcup \cdots \sqcup C_{l,m}$. Since they are strictly contained in W_l', by (a) they also belong to $C(T)$. Let $x_1, \ldots, x_m$ be the nodes in T with $C_T(x_i) = C_{l,i}$, for $i = 1, \ldots, m$. It turns out that each x_i is a child of w_l.
  Indeed, since $C_{l,i} \subsetneq C_l = W_l$, x_i is a proper descendant of w_l. If it is not its child, then the path $w_l \rightsquigarrow x_{l,i}$ contains an intermediate node $\bar{x}$ with cluster $C_{l,i} \sqcup X$, with $\emptyset \neq X \subsetneq C_l \setminus C_{l,i}$. Then, $C_{l,i} \sqcup X$ is also a cluster in T'. But in T' we have that x_i', with cluster $C_{l,i}$, is a child of w_l', with cluster $B \sqcup C_l$, and this leads to a contradiction, because $C_{l,i} \subsetneq C_{l,i} \sqcup X \subsetneq B \sqcup C_l$.

In summary, the structures of T and T' are those described in Figure 24. It is straightforward to check that the sets $A_1, \ldots, A_k, B, C_1, \ldots, C_{l-1}, C_{l,1}, \ldots, C_{l,m}$ satisfy the conditions in Proposition 6. □

References

1. Dress, A., Huber, K.T., Koolen, J., Moulton, V., Spillner, A.: Basic Phylogenetic Combinatorics. Cambridge University Press, Cambridge, UK (2013)
2. Aho, A.V., Sagiv, Y., Szymanski, T.G., Ullman, J.D.: Inferring a tree from lowest common ancestors with an application to the optimization of relational expressions. SIAM Journal on Computing **10**(3), 405–421 (1981)

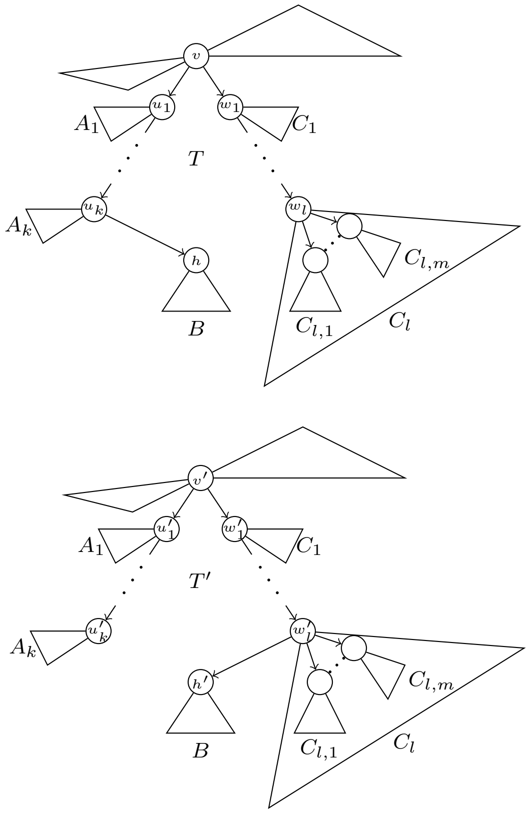

Figure 24 The structures of T and T' when they satisfy the conditions in Proposition 8.
